# Design, synthesis, and *in vitro* evaluation of BP-1-102 analogs with modified hydrophobic fragments for STAT3 inhibition

**DOI:** 10.1080/14756366.2020.1871336

**Published:** 2021-01-13

**Authors:** Patrik Oleksak, Miroslav Psotka, Marketa Vancurova, Olena Sapega, Jana Bieblova, Milan Reinis, David Rysanek, Romana Mikyskova, Katarina Chalupova, David Malinak, Jana Svobodova, Rudolf Andrys, Helena Rehulkova, Vojtech Skopek, Pham Ngoc Lam, Jiri Bartek, Zdenek Hodny, Kamil Musilek

**Affiliations:** a Department of Chemistry, Faculty of Science, University of Hradec Kralove, Hradec Kralove, Czech Republic; b Department of Genome Integrity, Institute of Molecular Genetics of the Czech Academy of Sciences, Prague, Czech Republic; c Laboratory of Immunological and Tumour Models, Institute of Molecular Genetics of the Czech Academy of Sciences, Prague, Czech Republic; d Genome Integrity Unit, Danish Cancer Society Research Center, Copenhagen, Denmark

**Keywords:** STAT3 signalling pathway, cancer, SH2 domain, inhibitor, structure–activity relationship

## Abstract

Twelve novel analogs of STAT3 inhibitor BP-1-102 were designed and synthesised with the aim to modify hydrophobic fragments of the molecules that are important for interaction with the STAT3 SH2 domain. The cytotoxic activity of the reference and novel compounds was evaluated using several human and two mouse cancer cell lines. BP-1-102 and its two analogs emerged as effective cytotoxic agents and were further tested in additional six human and two murine cancer cell lines, in all of which they manifested the cytotoxic effect in a micromolar range. Reference compound S3I-201.1066 was found ineffective in all tested cell lines, in contrast to formerly published data. The ability of selected BP-1-102 analogs to induce apoptosis and inhibition of STAT3 receptor-mediated phosphorylation was confirmed. The structure–activity relationship confirmed a demand for two hydrophobic substituents, i.e. the pentafluorophenyl moiety and another spatially bulky moiety, for effective cytotoxic activity and STAT3 inhibition.

## 1. Introduction

Signal transducers and activators of transcription (STAT) signalling pathways belong to signalling modules that are relatively simple and straightforward: upon appropriate stimuli (binding of a polypeptide-based ligand), a plasma membrane receptor-associated kinase phosphorylates, and thus activates (without use of a second messenger) transcription factors of the STAT family to execute a specific biological function by regulating gene expression. Human STAT transcription factors (STAT1, STAT2, STAT3, STAT4, STAT5a, STAT5b, and STAT6) are involved in the response to autocrine or paracrine stimuli in a multitude of cellular functions such as regulation of cell proliferation, apoptosis, differentiation, and stress response^1–3^.

The STAT3 protein structure is characterised by four domains involved in oligomerisation, binding to DNA, and transactivation activity, and a Src homology 2 (SH2) domain mediating phosphorylation-dependent dimerisation. The SH2 domain contains tyrosine 705 (Y705), which is phosphorylated in response to extracellular signalling by kinases of the Jak and Src families. STAT3 proteins with phosphorylated Y705 (pY705) form dimers via reciprocal binding of SH2 domains and they are further translocated into the nucleus to bind to specific DNA response elements. STAT3 are both activators and repressors of hundreds of genes, including cell cycle regulators *MYC* and *CCND1* and anti-apoptotic BCL-2 family genes *BCL2*, *BCL2L2* (BCL-W), *BCL2L1* (BCL-XL), *MCL1*, and *BIRC5* (survivin; reviewed in refs.^4^^,^^5^).

The signalling pathway executed by STAT3 (hence the STAT3 signalling pathway) attracts specific attention of pharmaceutical research due to its involvement in human inflammatory and malignant diseases. Under normal conditions, activation of the STAT3 signalling pathway is transient due to a number of negative regulators. However, STAT3 signalling was found to be constitutively activated in several solid and haematological malignancies (e.g. refs.^6^^,^^7^). In effect, STAT3 signalling contributes to all hallmarks of cancer as defined by Hanahan and Weinberg^8^, including aberrant cell proliferation, regulation of cell death, tumour-promoting inflammation, invasion and metastasis, immunosuppression, genome instability, tumour metabolism, and treatment resistance^4^^,^^5^^,^^9^^,^^10^. Constitutive STAT3 activation is associated with worse prognosis, for instance, of patients with gliomas^11^^,^^12^. Due to this functional role in malignant progression, the inhibition of activity of STAT3 signalling is considered a potential target for cancer treatment^13^^,^^14^.

Current approaches to inhibiting the STAT3 signalling pathway focus on several levels of the signalling module from inhibition of upstream activating kinase Jak1, inhibition of SH2 domain phosphorylation and dimerisation, to inhibition of the transcription-activating domain and DNA-binding activity^15–22^. However, despite intensive efforts, currently there is no STAT3 inhibitor in clinical practice. As summarised by Huang et al., the reasons for that are multiple. The most unfavourable for development of inhibitors targeting the SH2 domain is the fact that the pY705-peptide binding site within the STAT3 SH2 domain spreads over large, relatively flat surfaces and lacks well-defined deep binding pockets. It makes the specific binding of the small molecular inhibitors difficult. In addition, several direct STAT3 inhibitors are also characterised by low aqueous solubility, low cell permeability and poor oral bioavailability, which may hinder the drug development and clinical studies^23^.

Among the small molecules, BP-1-102 was formerly found to be a direct STAT3 inhibitor with reasonable *in vivo* tumour-inhibiting activity and commercial availability. It was synthesised as an analogue of S3I-201.1066 and it is proposed to bind specifically to the STAT3 SH2 domain^24^.

In this study, novel analogs of commercially available STAT3 inhibitor BP-1-102 (**1**; Table 1) were designed, synthesised, and their cytotoxic activity plus receptor-mediated STAT3 phosphorylation inhibitory ability were determined.

**Table 1.** Prepared final products **1**–**14**.

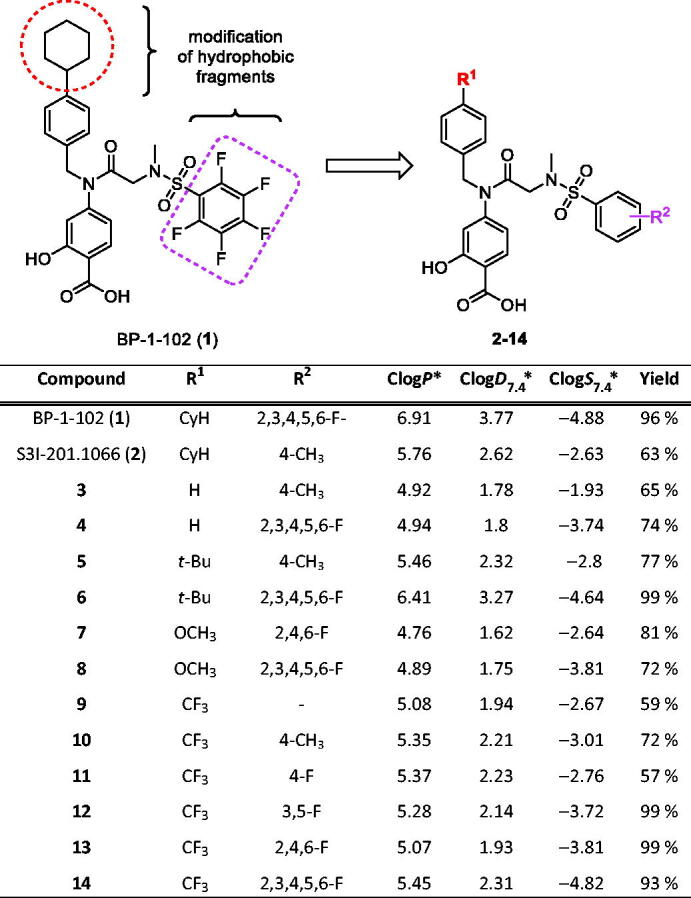	

^a^ Clog*P*, Clog*D*_7.4_, and Clog*S*_7.4_ were calculated in ACDLabs PhysChemSuite 14.0.

## 2. Results and discussion

### 2.1. Molecular design and prediction of physical–chemical properties

BP-1-102 (**1**) was found to be an effective inhibitor of STAT3 both *in vitro* and *in vivo*^24^, but with limited solubility in water/buffer compartments. Therefore, it is possible to modify the hydrophobic substituents by other moieties to plausibly decrease the hydrophobicity of the molecule and enhance the water/buffer solubility, while at the same time evaluating the antitumor efficacy (Table 1). First, the cyclohexylbenzyl moiety can be omitted or replaced by less bulky hydrophobic fragments (e.g. *t*-butyl, methoxy, and trifluoromethyl). Additionally, the pentafluorophenyl moiety can be replaced by a tolyl fragment (similarly as in the effective STAT3 inhibitor S3I-201.1066^25^, **2**) or by mono-, di-, or trifluorophenyl with very similar steric/spatial properties.

The physical–chemical properties (log*P*, log*D*_7.4_, and log*S*_7.4_) of the designed molecules were predicted to depict differences from the parent molecule **1** (Table 1). Using the proposed changes in hydrophobic substituents, the log*P* values were usually decreased compared to standards **1** or **2**, reaching the optimal range for barrier penetrability (∼5)^25^ with the exception of molecules **5**, **6**, **10**–**12**, and **14**. In case of log*D*, the values were decreased when compared to standards, again with the exception of molecules **5**, **6**, **10**–**12**, and **14**, suggesting lower lipid binding. Concerning the solubility prediction (log*S*_7.4_), the majority of the proposed molecules, except for molecules **10** and **14**, should be at least slightly more soluble than the standard **1**.

### 2.2. Synthesis of novel compounds

**Scheme 1.**
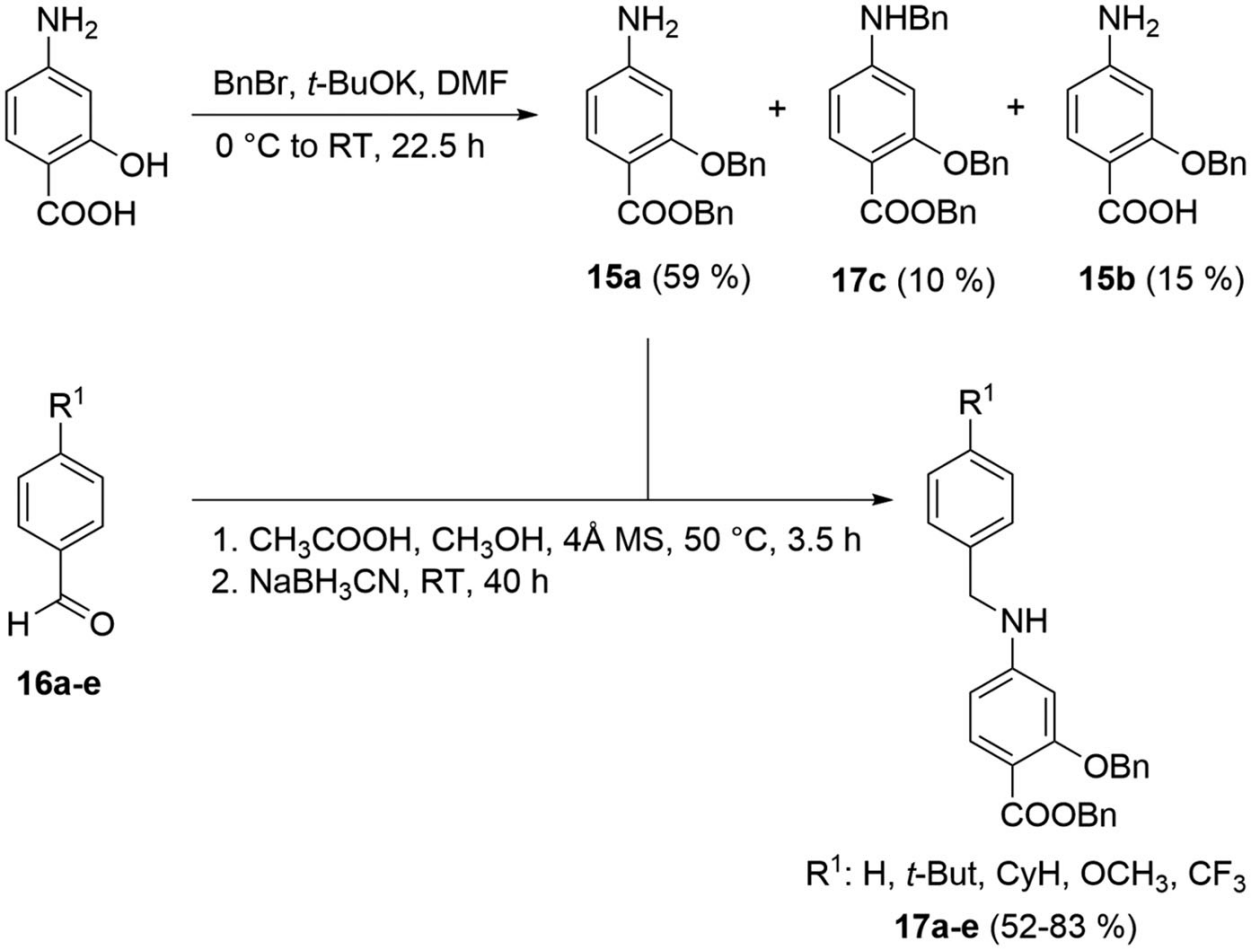
Preparation of secondary amines **17a**–**e** as the first coupling partners.

The synthetic strategy towards preparation of standards **1**–**2** and designed *p*-aminosalicylic acid sulphonamides **3**–**14** was based on the methodology reported by Page et al.^26^ The convergent synthesis involved two main routes. Initial benzylation of *p*-aminosalicylic acid with BnBr and *t*-BuOK in DMF gives 59% yield of desired dibenzylated derivative **15a**, with tribenzylated **17c** and mono-*O*-benzylated **15b** byproducts (Scheme 1). Dibenzylated amine **15a** was used for direct condensation with selected *p*-substituted benzaldehydes **16a**–**e** with subsequent reductive amination. These conditions allowed preparation of a series of substituted secondary amines **17a**–**e**, which represent the first coupling partners for amide reaction. Substituted aldehyde **16a** was prepared according to formerly reported procedures^27^, other aldehydes **16b**–**e** were commercially purchased.

**Scheme 2.**
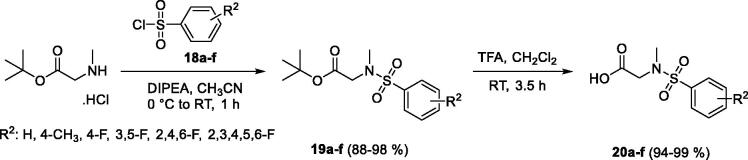
Preparation of substituted carboxylic acids **20a**–**f** as the second coupling partners.

Sarcosine *t*-butyl ester hydrochloride was used as the starting material for preparation of the carboxylic coupling partner (Scheme 2). Sarcosine ester in reaction with selected benzenesulfonyl chlorides **18a**–**f** provided corresponding sulphonamides **19a**–**f** in good to excellent yields. The following acid hydrolysis promoted with TFA in CH_2_Cl_2_ allowed preparation of desired substituted sulphonamide carboxylic acids **20a**–**f** in excellent yields. The substituted carboxylic acids **20a**–**f** were the second partners for the amide coupling reaction.

**Scheme 3.**
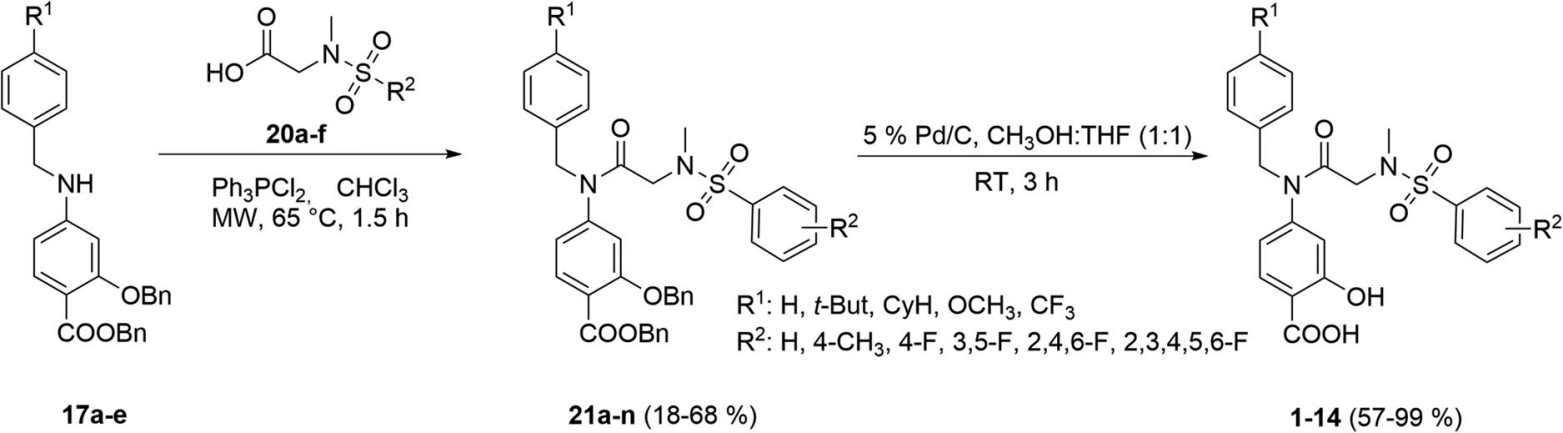
Amide coupling and deprotection leading to final products **1**–**14**.

The microwave-assisted amide coupling^28^ between substituted secondary amines **17a**–**e** and substituted carboxylic acids **20a**–**f** was promoted with Ph_3_PCl_2_ and represents the key reaction step^29^^,^^30^. Although yields of the coupling reactions were highly variable (Table 1), provided amides **21a**–**n** were the direct precursors of the final products. Thus, final debenzylation catalysed with 5% Pd/C in CH_3_OH/THF (1:1) yielded target *p*-aminosalicylic acid analogs **1**–**14** (Scheme 3). The structure of the final products was characterised by NMR (Figures S1–S14) and HRMS analysis. The purity was estimated using the HPLC method and was found to be >95% for all final products.

### 2.3. *In vitro* screening of cytotoxic effects

**Figure 1.**
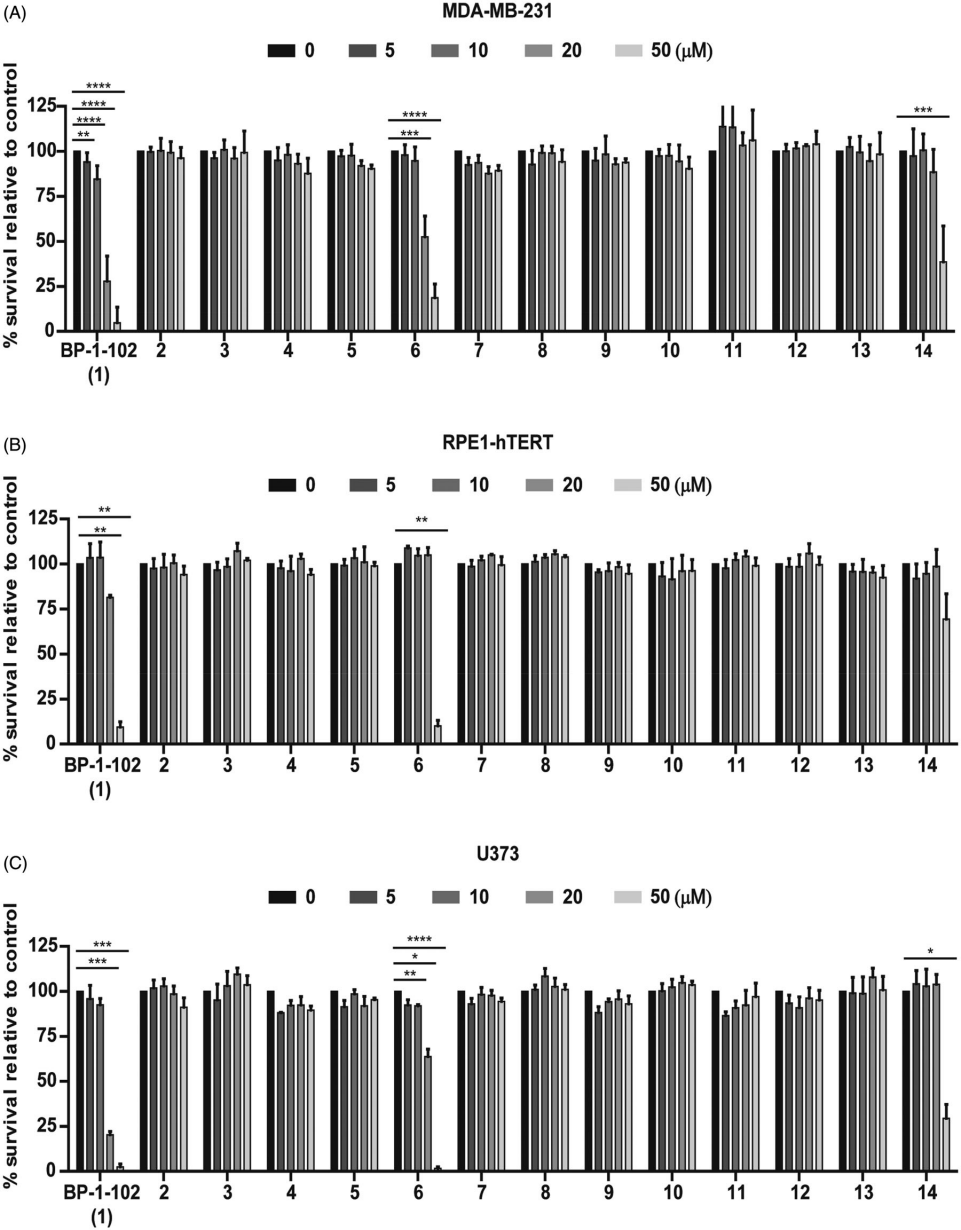
The cytotoxic effect of newly synthesised compounds (concentration range 0–50 µM, 24 h) on breast cancer MDA-MB-231 (A), immortalised RPE-1 (B), and glioblastoma U373 (C) cell lines was tested by the resazurin assay (A) or crystal violet staining (B, C). Data were normalised to control/untreated samples and plotted as mean ± SD (*n* ≥ 3).

Using BP-1-102 (**1**) and S3I-201.1066 (**2**) as reference compounds with STAT3 inhibitory activity^24^^,^^31^, the cytotoxic effects of 12 newly prepared BP-1-102 analogs (**3**–**14**) were compared using the human breast cancer MDA-MB-231 cell line with constitutive STAT3 signalling routinely used for testing of chemically induced STAT3 inhibition^32^^,^^33^. The cells were treated with the compounds at concentrations 0, 5, 10, 20, and 50 µM for 24 h, and the cytotoxicity was estimated by the resazurin assay^34^. Apart from the compounds **3**, **7**–**8**, and **10**–**13** lacking any significant cytotoxicity, the compounds **4** and **5** showed mild-to-negligible cytotoxicity in the tested concentration range. Note that the reference compound S3I-201.1066 (**2**) did not show any observable cytotoxic effect in contrast to published data^31^. However, the compounds **6** and **14** were cytotoxic starting from concentration 10 µM and 20 µM, respectively (Figure 1(A)). Similar results were obtained with human immortalised retinal pigment epithelium RPE-1 (Figure 1(B)) and human glioblastoma U373 (Figure 1(C)) cell lines, which were exposed to BP-1-102 and to the novel analogs and analysed by the crystal violet assay. Again, compounds BP-1-102 (**1**), **6** and **14** showed concentration-dependent cytotoxic activity.

To complement the resazurin assay, which estimates cell metabolic activity as an indirect measure of the compound cytotoxicity, cell death induction was confirmed by annexin V and propidium iodide (PI) staining for apoptosis/necrosis and analysis by fluorescence-activated flow cytometry (FACS) in MDA-MB-231 cells exposed to BP-1-102 (**1**), **6** and **14** at concentrations 0, 5, 10, 20, and 50 µM for 24 h (Figure S15A–B).

To estimate IC_50_ of **6** and **14**, MDA-MB-231 cells were treated with **1** (0, 2, 5, 10, 15, 20, 25, 30, 40, and 50 µM), **6** (0, 5, 10, 20, 25, 30, 40, 50, 70, and 100 µM), and **14** (0, 20, 40, 50, 60, 70, 80, 100, 150, and 200 µM) for 24 h. Cytotoxicity was estimated by the resazurin assay, and IC_50_ was calculated using a nonlinear regression module in GraphPad Prism (GraphPad Software, La Jolla, CA) as 14.96 µM for BP-1-102 (**1**), 26.21 µM for **6**, and 39.30 µM for **14** (Figure S15C). Thus, the compound **6** showed comparable inhibitory activity as the parent molecule BP-1-102 (**1**). Note that the IC_50_ of BP-1-102 agreed with the previously estimated values^24^.

**Figure 2.**
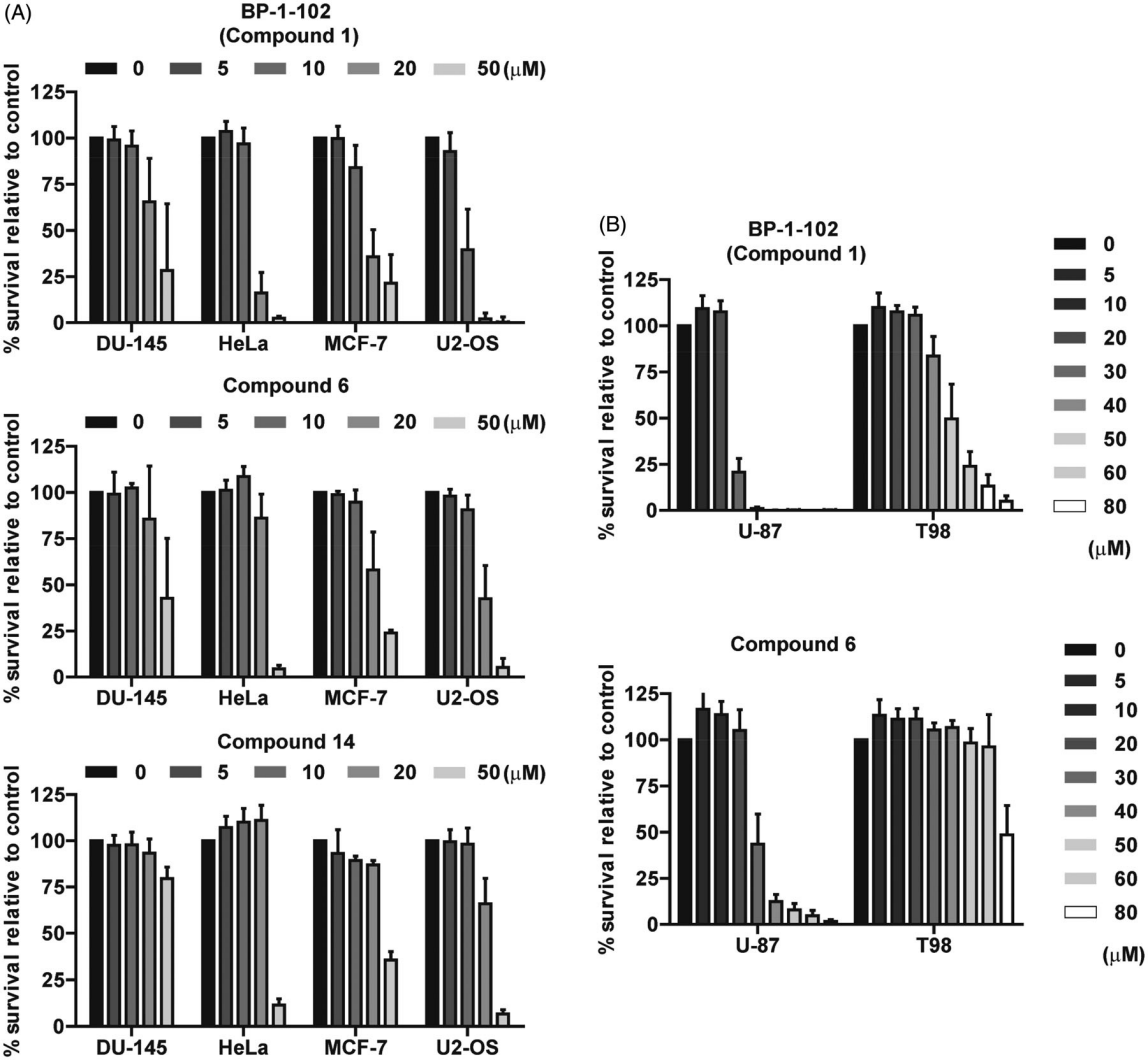
(A) The cytotoxic effect of BP-1-102 (**1**) and newly synthesised compounds **6** and **14** (concentration range 0–50 µM, 24 h) was tested on human prostate carcinoma DU-145, cervix carcinoma HeLa, breast adenocarcinoma MCF-7, and osteosarcoma U2-OS cell lines by the resazurin assay. (B) The cytotoxic effect of BP-1-102 (**1**) and newly synthesised compound **6** was tested on human glioblastoma U-87 and T98 cell lines using crystal violet staining. Data were normalised to control/untreated samples and plotted as mean ± SD (*n* = 3).

Further, the cytotoxicity and possible cell type-dependent differences of the effective BP-1-102 analogs were tested in human cancer cells of various origin. Cell lines derived from several human solid malignancies, namely, human breast adenocarcinoma MCF-7, cervix carcinoma HeLa, prostate carcinoma DU-145, and osteosarcoma U2-OS, were treated with the most potent compound **6** at concentrations 0, 5, 10, 20, and 50 µM for 24 h, and cytotoxicity was estimated by the resazurin assay. As shown in Figure 2(A), all tested cell lines were sensitive to BP-1-102 (**1**) to a comparable extent; nevertheless, HeLa and U2-OS cells were relatively more sensitive to BP-1-102 (**1**) compared to cells with constitutive STAT3 activity DU-145 and MCF-7. Compound **6** in the DU-145, HeLa, MCF-7, and U2-OS cells manifested a similar or slightly lower cytotoxic effect as the parent molecule BP-1-102 (**1**). Furthermore, the cytotoxicity of compound **6** was compared to BP-1-102 (**1**) in human glioblastoma cell lines U-87 and T98 in concentration range 0–80 µM for 24 h using the crystal violet assay. T98 cells, one of the most chemoresistant human glioblastoma cell lines, showed remarkable resistance to both BP-1-102 (**1**) and **6** when compared to U-87 cells (Figure 2(B)). Altogether, the novel analogue **6** manifested similar cytotoxic activity for human cancer cells as parental compound BP-1-102.

The less potent compound **14** was tested in four human cancer cell lines DU-145, HeLa, MCF-7, and U2-OS. Although the cytotoxic effect on HeLa, MCF-7, and U2-OS was comparable to BP-1-102 (**1**) and compound **6** at 50 µM, it was markedly decreased at lower concentrations, and the prostate carcinoma DU-145 cell line exhibited a weaker response to compound **14** (Figure 2(A)).

**Figure 3.**
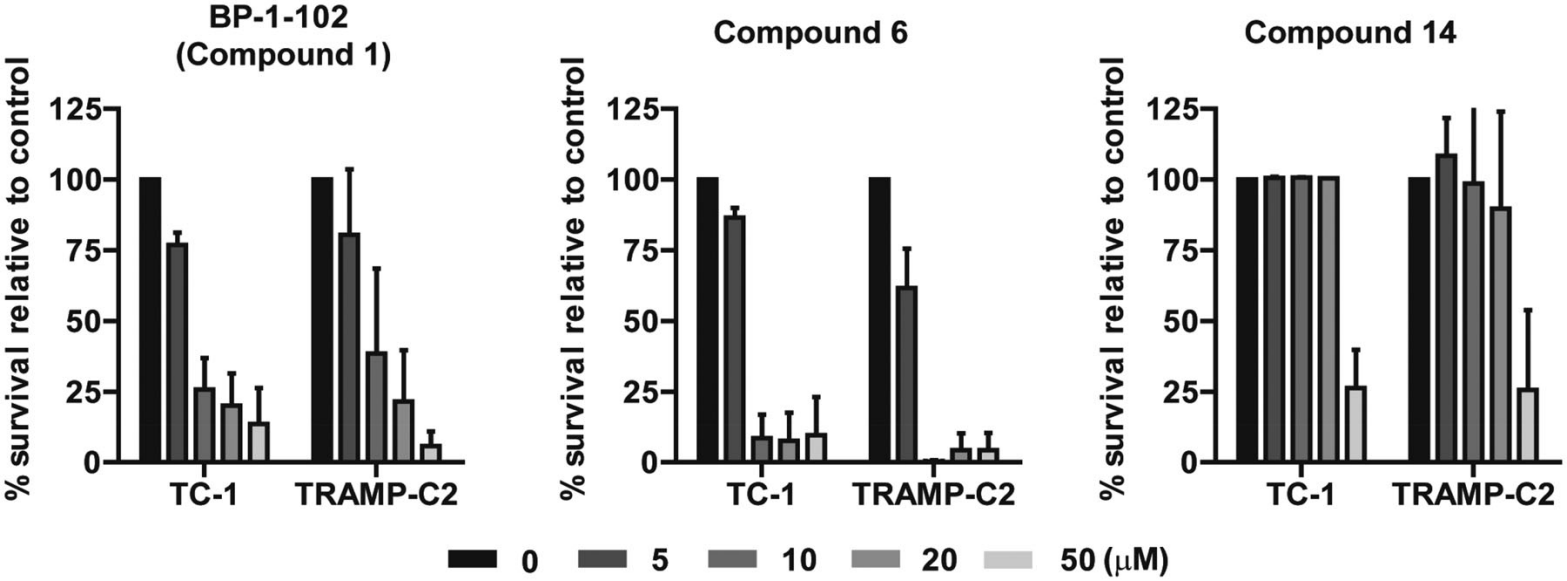
The cytotoxic effect of BP-1-102 (**1**) and newly synthesised compounds **6** and **14** (concentration range 0–50 µM, 24 h) was tested on mouse TC-1 lung and TRAMP-C2 prostate cancer cell lines by the MTT assay. Data were normalised to control/untreated samples and plotted as mean ± SD (*n* = 2).

To uncover the possible differences between mouse vs. human cells, the whole set of compounds was screened in mouse lung TC-1 and prostate TRAMP-C2 cancer cell lines using the MTT assay (Figure S16). Similarly to human cells, compound **2** (S3I-201.1066) did not demonstrate any cytotoxic effect in both murine cell lines, whereas compounds **6** and **14** displayed cytotoxic activity comparable to BP-1-102 (**1**) (Figure 3) with IC_50_ summarised in Table 2. Additionally and in contrast to human cells, compound **8** manifested a mild cytotoxic effect. Further, the cytotoxicity of the selected compounds **1**, **6**, and **14** in murine cells was also confirmed by annexin V and PI staining for cell apoptosis/necrosis and by FACS analysis (Figures S17–S18).

**Table 2.** Antiproliferative activity of the tested compounds.

Compound	R^1^	R^2^	IC_50_±SD (µM)^a^				IC_50_±SD (µM)^b^	
			MDA-MB-231	DU-145	MCF-7	RPE-1	TC-1	TRAMP-C2
BP-1-102 (**1**)	CyH	2,3,4,5,6- F	15.0 ± 3.7	18.0 ± 8.3	–	13.9 ± 0.2	7.8 ± 1.1	7.9 ± 4.5
S3I-201.1066 (**2**)	CyH	4-CH_3_	–	–	–	–	–	–
**3**	H	4-CH_3_	–	–	–	–	–	–
**4**	H	2,3,4,5,6-F	>50	>50	>50	>50	–	–
**5**	*t*-Bu	4-CH_3_	–	–	–	–	–	–
**6**	*t*-Bu	2,3,4,5,6-F	26.2 ± 5.6	34.0 ± 2.6	19.6 ± 3.6	24.5 ± 2.6	6.2 ± 1.1	14.50 ± 13.40
**7**	OCH_3_	2,4,6-F	–	–	–	–	–	–
**8**	OCH_3_	2,3,4,5,6-F	–	–	–	–	–	–
**9**	CF_3_	–	–	–	–	–	–	–
**10**	CF_3_	4-CH_3_	–	–	–	–	–	–
**11**	CF_3_	4-F	–	–	–	–	–	–
**12**	CF_3_	3,5-F	–	–	–	–	–	–
**13**	CF_3_	2,4,6-F	–	–	–	–	–	–
**14**	CF_3_	2,3,4,5,6-F	39.3 ± 6.3	65.3 ± 7.8	47.3 ± 3.8	39.7 ± 7.4	25.3 ± 6.6	23.1 ± 10.5

^a^ The inhibitory effect of the compounds on the proliferation of four human cell lines was determined by the resazurin assay: SD – standard deviation; data are the mean ± SD from at least two independent experiments.

^b^ The inhibitory effect of the compounds on the proliferation of two mouse cell lines was determined by the MTT assay: SD – standard deviation; data are the mean ± SD from at least two independent experiments.

### 2.4. Inhibitory effect on STAT3 phosphorylation

**Figure 4.**
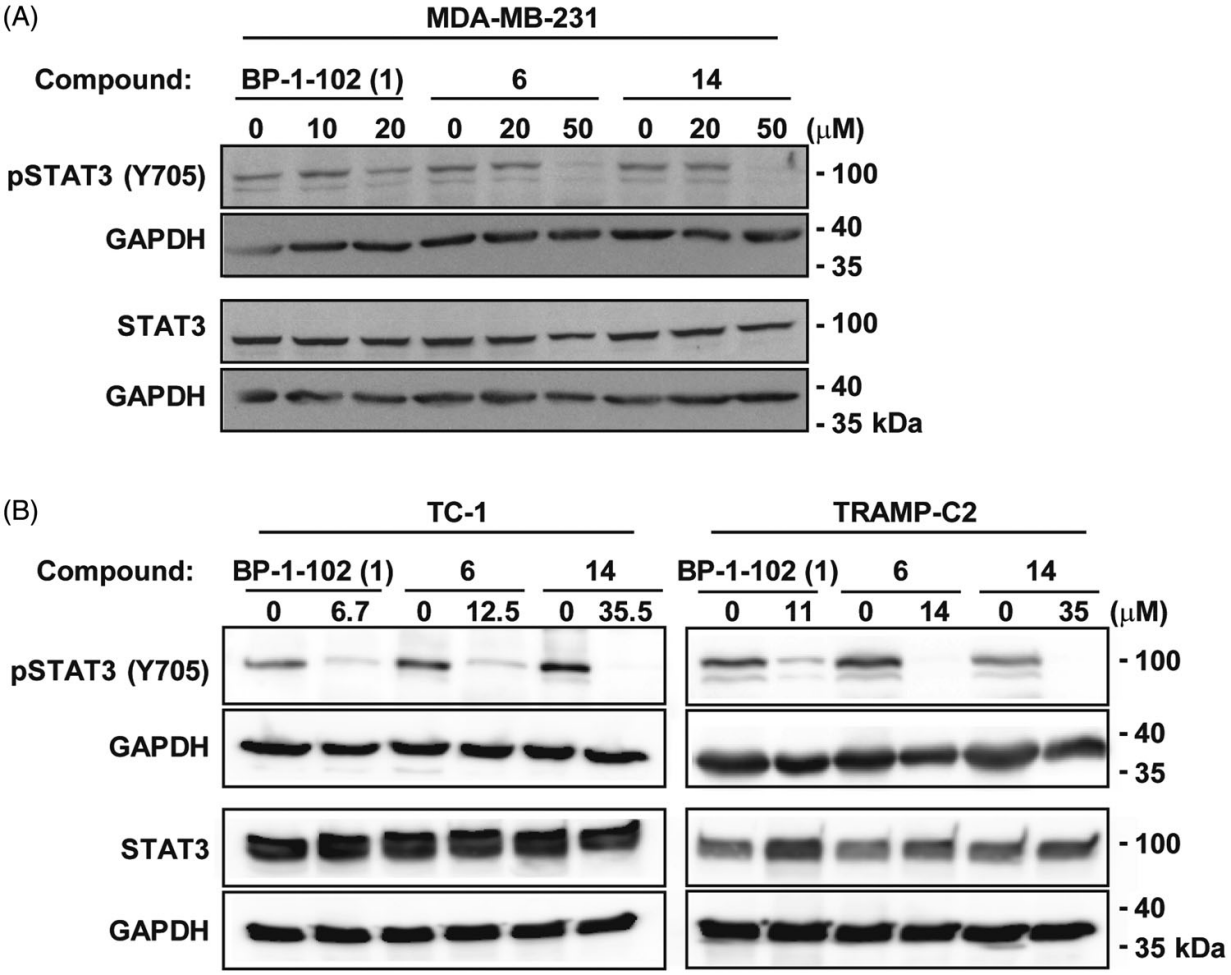
Human MDA-MB-231 (A) or mouse TC-1 and TRAMP-C2 (B) cells were treated with BP-1-102 (**1**) and two newly synthesised compounds **6** and **14** at the given concentrations for 4 h. Whole cell lysates were subjected to SDS-PAGE/immunoblotting analysis and probed for pSTAT3 (Y705) and total STAT3. GAPDH was used as a loading control.

The inhibitory effect of the selected compounds on receptor (JAK)-mediated phosphorylation of STAT3 were demonstrated in MDA-MB-231 cells with constitutive STAT3 Y705 phosphorylation (pY705). The cells were exposed to BP-1-102 (**1**), compounds **6** and **14** in a micromolar concentration range for 4 h, and then the levels of STAT3 pY705 and total STAT3 were probed by immunoblotting with specific antibodies. As shown in Figure 4(A), both the reference and two novel compounds inhibited constitutive STAT3 phosphorylation. Similar effects of all three compounds were also observed using mouse cell lines TC-1 and TRAMP-C2 (Figure 4(B); cytotoxic concentrations of individual drugs used in these experiments were derived from annexin V/PI apoptosis analysis; see Figures S17–S18). It can be concluded that both novel compounds **6** and **14** manifested inhibitory effects on receptor-mediated phosphorylation of Y705 of STAT3 in both human and mouse cell lines.

### 2.5. Structure–activity relationship

**Figure 5.**
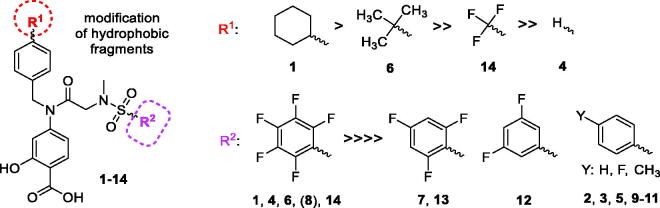
Structure–activity relationship for the cytotoxicity of BP-1-102 and its analogs (**1**–**14**).

The structure–activity relationship was based on chemical modification made in both hydrophobic regions and its correlation to cytotoxic effects of the studied molecules. First, the cyclohexyl moiety omission led to a decrease of cytotoxic activity (**1**>**4**), i.e. increased IC_50_ values in the selected cell lines (Table 2; Figure 5). The replacement of cyclohexyl by *t*-butyl or trifluoromethyl resulted in higher cytotoxicity (decreased IC_50_) for all tested cell lines, and spatially bulkier *t*-butyl was found twofold more potent than trifluoromethyl, but still less effective than the original cyclohexyl moiety (**1**>**6**>**14**).

Additionally, replacement of the pentafluorophenyl moiety by mono-, di-, or trifluorophenyl led to complete loss of cytotoxic activity irrespective of changes of the R^1^ substituent in compounds **2**–**3**, **5**, **7**, **9**, and **10**–**13**. Only one compound with the intact pentafluorophenyl group (**8**) showed no cytotoxic activity in human MDA-MB-231 and U373 cells, while mild cytotoxicity in murine cells (Figure S16), indicating that the pentafluorophenyl group highly contributes to the cytotoxic effect of the BP-1-102-derived scaffold. Interestingly, our data obtained with compound S3I-201.1066 (**2**) differ from the previously published studies. S3I-201.1066 (**2**) was reported to affect viability of human breast cancer MDA-MB-231 and pancreatic cancer Panc-1 cell lines^31^. Although our findings do not support that, as compound **2** did not show any cytotoxic effect, they are still in line with the importance of the pentafluorophenyl moiety for cytotoxic activity and STAT3 inhibition.

Concerning prediction of the physical–chemical parameters, the molecules (**1**, **6**, **14**) with increased log*P*, log*D*_7.4_ and decreased log*S*_7.4_ were found to have better cytotoxic activity, presuming these compounds to be well penetrable across the membranes, but less soluble in the water/buffer environment. Concerning the cytotoxic activity overall, hydrophobic substituents with an optimal pentafluorophenyl moiety in the sulphonamidic part of the molecule and a spatially bulky moiety in the benzyl part of the molecule are important prerequisites for the biological effect of the compound.

## 3. Conclusions

Twelve novel analogs of STAT3 inhibitor BP-1-102 were designed with the aim to modify hydrophobic fragments of the molecule, and these compounds were successfully synthesised. The cytotoxic activity of reference compounds BP-1-102 (**1**) or S3I-201.1066 (**2**) and the novel compounds was screened using two human and two mouse cancer cell lines, and two potent inhibitors (**6**, **14**) were selected. The selected compounds were further tested in detail in other six human and two murine cancer cell lines, in all of which they manifested the cytotoxic effect in a micromolar range. Their ability to induce apoptosis and inhibition of STAT3 receptor-mediated phosphorylation was confirmed. Notably, compound S3I-201.1066 (**2**) emerged as ineffective for the growth inhibition of the used cell lines. Importantly, it was found that glioblastoma cell line T98, one of the most chemoresistant glioblastoma cell lines, is also resistant to BP-1-102 (**1**) and its most effective analogue in this study, compound **6**. The structure–activity relationship confirmed the demand for two hydrophobic substituents, i.e. the pentafluorophenyl moiety and the second spatially bulky moiety for effective cytotoxic activity and STAT3 inhibition.

## 4. Experimental

### 4.1. General synthetic methods

All used commercial reagents and solvents were purchased in the highest available purity from supplier Sigma-Aldrich (St. Louis, MO). Prepared compounds were purified by column chromatography on silica gel Kieselgel 60 (0.040–0.063 mm, 230–400 mesh, Merck, Kenilworth, NJ). Thin layer chromatography was performed on Merck silica gel 60 F_254_ analytical plates, using a mobile phase corresponding to the mobile phase used for the glass column chromatography. Structures on TLC were detected using either UV light (254 nm) or spraying with the detection reagent (10% solution of phosphomolybdic acid in MeOH) with subsequent heating. Microwave-assisted reactions were accomplished in a focussed Discover Microwave System (CEM Corporation, Stallings, NC), and the contents of the vessel were cooled down rapidly by a stream of compressed air. Melting points of solid compounds were recorded in a Melting Point Apparatus-Büchi M-565 and were uncorrected. Electrosprayionisation mass spectrometry (ESI-MS) was evaluated in an Agilent 6470 Triple Quadrupole mass spectrometer (Agilent Technologies, Palo Alto, CA) in a positive or negative mode. High resolution mass spectrometry (HRMS) was determined by a Q Exactive Plus hybrid quadrupole-orbitrap spectrometer (ThermoFisher, Bremen, Germany). NMR spectra were recorded in CD_3_OD-d_4_, CDCl_3_-d, or DMSO-d_6_ solution at ambient temperature in a Varian S500 spectrometer (499.87 MHz for ^1^H and 125.71 MHz for ^13^C). Chemical shifts were recorded as *δ* values in parts per million (ppm), were referred to solvent signal (CD_3_OD-d_4_: 3.31 ppm for ^1^H and 49.00 ppm for ^13^C; CDCl_3_-d: 7.26 ppm for ^1^H and 77.16 ppm for ^13^C; DMSO-d_6_: 2.50 ppm for ^1^H and 39.52 ppm for ^13^C), and spin multiplicities are given as s (singlet), d (doublet), dd (doublet of doublets), t (triplet), tt (triplet of triplets), or m (multiplet). Coupling constants (*J*) are given in hertz (Hz). The purity of all the final products was ≥92%, based on LC with UV detection (*λ* = 254 nm).

### 4.2. Chemical synthesis and characterisation of the compounds

#### 4.2.1. General procedure for benzylation (15a–b)

*t*-BuOK (2.418 g; 21.55 mmol) was added to *p*-aminosalicylic acid (3000 g; 19.59 mmol) dissolved under N_2_ atmosphere in dry DMF (103 ml; 5.26 ml/mmol) cooled to 0 °C. After 15 min, BnBr (2.56 ml; 21.55 mmol) was added dropwise, and the mixture was stirred for another 15 min at 0 °C. The mixture was then allowed to warm to RT and stirred for 5.5 h. Further, the reaction mixture was again cooled down to 0 °C and a second portion of *t*-BuOK (2.418 g; 21.55 mmol) was added. After 15 min, a second portion of BnBr (2.56 ml; 21.55 mmol) was added dropwise to the reaction. The mixture was stirred for another 15 min at 0 °C then allowed to warm to RT and stirred for 17 h. The reaction mixture was cooled to 0 °C, and then saturated aqueous solution of NaHCO_3_ (50 ml) and H_2_O (75 ml) were added. The mixture was transferred to a separatory funnel, and the organic product was extracted to ethyl acetate (EA) (3 × 20 ml). Organic layers were combined, washed with H_2_O (75 ml), then with saturated aqueous NaCl (40 ml), and dried with Na_2_SO_4_ and filtered. The organic solvent was removed under reduced pressure, and the residue was purified by column chromatography on silica gel in the mobile phase petrolether (PE):EA:Et_3_N (4:1:2%) to produce the desired product **15a** with tribenzylated **17c** and monobenzylated **15b** as byproducts.

*Benzyl 4-amino-2-(benzyloxy)benzoate* (**15a**). Isolated in 59% yield as a pale-yellow crystalline solid. Mp: 86.0 ± 1 °C; *δ*_H_ (500 MHz, DMSO-d_6_): 5.07 (2H, s, CH_2_(Bn)), 5.20 (2H, s, CH_2_(Bn)), 5.96 (2H, s, NH_2_), 6.19 (1H, dd, *J* = 8.6 Hz, *J* = 1.9 Hz, Ar), 6.32 (1H, d, *J* = 1.9 Hz, Ar), 7.28–7.39 (8H, m, Ar), 7.46–7.49 (2H, m, Ar), 7.60 (1H, d, *J* = 8.6 Hz, Ar); *δ*_C_ (126 MHz, DMSO-d_6_): 64.8, 69.1, 97.6, 105.5, 105.9, 127.0, 127.4, 127.6, 127.7, 128.2, 128.3, 133.7, 137.0, 137.1, 154.7, 160.4, 164.9; ESI-MS [M + H]^+^ calculated for C_21_H_20_NO_3_^+^ (*m*/*z*): 334.1, found 334.2.

*4-Amino-2-benzyloxybenzoic acid* (**15b**). Isolated in 15% yield as a pale-orange crystalline solid. Mp: 140.0 ± 1 °C; *δ*_H_ (500 MHz, DMSO-d_6_): 5.29 (2H, s, CH_2_(Bn)), 6.01 (1H, d, *J* = 2.2 Hz, Ar), 6.13 (1H, dd, *J* = 8.8 Hz, *J* = 2.2 Hz, Ar), 6.16 (2H, bs, NH_2_), 7.31–7.36 (1H, m, Ar), 7.38–7.45 (4H, m, Ar), 7.49 (1H, d, *J* = 8.8 Hz, Ar), 10.7 (1H, s, COOH); *δ*_C_ (126 MHz, DMSO-d_6_): 66.3, 100.8, 106.9, 128.2, 128.4, 128.7, 131.8, 136.0, 153.6, 163.8, 169.9; ESI-MS [M + H]^+^ calculated for C_14_H_14_NO_3_^+^ (*m*/*z*): 244.1, found 244.2.

#### 4.2.2. General procedure for preparation of substituted secondary amine (17a–e)

To a stirred solution of corresponding aldehyde **16a**–**e** (1.70 mmol) in anhydrous MeOH (13.3 ml; 13.3 ml/mmol) under N_2_ atmosphere, 4 Å molecular sieves were added followed by acetic acid (0.10 ml; 1.80 mmol). The mixture was stirred for 1 h at RT, the dibenzylated amine **15a** (1 mmol) was added, and the mixture was heated to 50 °C for 3.5 h. The reaction mixture was cooled to RT, NaBH_3_CN (157 mg; 2.50 mmol) was added, and the mixture was stirred for 40 h. The solvent was removed under reduced pressure, the residue was dissolved in CH_2_Cl_2_, filtered through a glass frit, and concentrated under reduced pressure. The organic phase was transferred to a separatory funnel, then saturated aqueous NaHCO_3_ (8 ml) and H_2_O (8 ml) were added for extraction. The aqueous layer was extracted with two portions of CH_2_Cl_2_ (both 8 ml). Combined organic layers were washed with H_2_O (12 ml) and brine (12 ml). After drying with Na_2_SO_4_, filtration and evaporation, the residue was purified by glass column chromatography on silica gel in mobile phase heptane:EA:Et_3_N (5:1:2%) to give corresponding secondary amine **17a**–**e**.

*Benzyl 2-(benzyloxy)-4-((4-cyclohexylbenzyl)amino)benzoate* (**17a**). Isolated in 73% as a white crystalline solid. Mp: 135.5 ± 1 °C; *δ*_H_ (500 MHz, CDCl_3_-d): 1.21–1.31 (1H, m, Cy), 1.35–1.46 (4H, m, Cy), 1.73–1.79 (1H, m, Cy), 1.81–1.91 (4H, m, Cy), 2.47–2.54 (1H, m, Cy), 4.27–4.31 (2H, m, CH_2_), 4.42 (1H, bs, NH), 5.08 (2H, s, CH_2_(Bn)), 5.31 (2H, s, CH_2_(Bn)), 6.17 (1H, d, *J* = 2.0 Hz, Ar), 6.21 (1H, dd, *J* = 8.6 Hz, *J* = 2.0 Hz, Ar), 7.19 (2H, d, *J* = 8.1 Hz, Ar), 7.24 (2H, d, *J* = 8.1 Hz, Ar), 7.26–7.35 (6H, m, Ar), 7.38–7.41 (2H, m, Ar), 7.43–7.46 (2H, m, Ar), 7.84 (1H, d, *J* = 8.6 Hz, Ar); *δ*_C_ (126 MHz, CDCl_3_-d): 26.3, 27.0, 34.6, 44.4, 47.7, 66.0, 70.6, 97.5, 105.1, 108.6, 127.1, 127.4, 127.7, 127.7, 127.9, 128.2, 128.5, 128.6, 134.5, 135.7, 137.0, 137.0, 147.7, 153.1, 161.1, 165.9; ESI-MS [M + H]^+^ calculated for C_34_H_36_NO_3_^+^ (*m*/*z*): 506.3, found 506.8.

*Benzyl 2-(benzyloxy)-4-((4-tert-butylbenzyl)amino)benzoate* (**17b**). Isolated in 83% as a pale-yellow crystalline solid. Mp: 91.0 ± 1 °C; *δ*_H_ (500 MHz, CDCl_3_-d): 1.33 (9H, s, *t*-Bu), 4.30 (2H, d, *J* = 5.3 Hz, CH_2_), 4.42 (1H, t, *J* = 5.3 Hz, NH), 5.09 (2H, s, CH_2_(Bn)), 5.31 (2H, s, CH_2_(Bn)), 6.17 (1H, d, *J* = 2.2 Hz, Ar), 6.22 (1H, dd, *J* = 8.6 Hz, *J* = 2.2 Hz, Ar), 7.24–7.34 (8H, m, Ar), 7.36–7.41 (4H, m, Ar), 7.43–7.46 (2H, m, Ar), 7.84 (1H, d, *J* = 8.6 Hz, Ar); *δ*_C_ (126 MHz, CDCl_3_-d): 31.5, 34.7, 47.6, 66.0, 70.6, 97.4, 105.1, 108.6, 125.8, 127.1, 127.4, 127.8, 128.2, 128.5, 128.6, 134.5, 135.4, 137.0, 137.0, 150.8, 153.1, 161.1, 165.9; ESI-MS [M + H]^+^ calculated for C_32_H_34_NO_3_^+^ (*m*/*z*): 480.2, found 480.4.

*Benzyl 4-(benzylamino)-2-(benzyloxy)benzoate* (**17c**). Isolated in 70% yield as a orange solid. Mp: 74.5 ± 1 °C; *δ*_H_ (500 MHz, DMSO-d_6_): 4.35 (2H, d, *J* = 6.0 Hz, CH_2_), 5.06 (2H, s, CH_2_(Bn)), 5.21 (2H, s, CH_2_(Bn)), 6.26 (1H, dd, *J* = 8.7 Hz, *J* = 1.9 Hz, Ar), 6.38 (1H, d, *J* = 1.9 Hz, Ar), 7.07–7.11 (1H, m, Ar), 7.22–7.27 (1H, m, Ar), 7.27–7.39 (11H, m, Ar), 7.43–7.46 (2H, m, Ar), 7.63 (1H, d, *J* = 8.7 Hz, Ar); *δ*_C_ (126 MHz, DMSO-d_6_): 46.0, 64.9, 69.3, 96.5, 104.6, 105.9, 126.9, 127.1, 127.3, 127.5, 127.6, 127.7, 128.0, 128.2, 128.3, 128.4, 133.5, 137.0, 137.0, 153.9, 160.3, 164.8; ESI-MS [M + H]^+^ calculated for C_28_H_26_NO_3_^+^ (*m*/*z*): 424.2, found 424.3.

*Benzyl 2-(benzyloxy)-4-((4-trifluoromethylbenzyl)amino)benzoate* (**17d**). Isolated in 52% yield as a yellow crystalline solid. Mp: 88.0 ± 1 °C; *δ*_H_ (500 MHz, CDCl_3_-d): 4.40 (2H, d, *J* = 5.7 Hz, CH_2_), 4.59 (1H, t, *J* = 5.7 Hz, NH), 5.06 (2H, s, CH_2_(Bn)), 5.30 (2H, s, CH_2_(Bn)), 6.12 (1H, d, *J* = 2.0 Hz, Ar), 6.18 (1H, dd, *J* = 8.6 Hz, *J* = 2.0 Hz, Ar), 7.27–7.34 (6H, m, Ar), 7.37–7.42 (6H, m, Ar), 7.58 (2H, d, *J* = 8.2 Hz, Ar), 7.82 (1H, d, *J* = 8.6 Hz, Ar); *δ*_C_ (126 MHz, CDCl_3_-d): 47.3, 64.6, 66.1, 70.6, 97.7, 105.1, 109.3, 125.6 (q, *J* = 3.8 Hz), 125.8 (q, *J* = 3.7 Hz), 127.0, 127.4, 127.8, 127.9, 128.2, 128.6, 128.6, 129.9 (q, *J* = 32.3 Hz), 134.5, 136.9, 142.7, 152.6, 161.0, 165.8; ESI-MS [M + H]^+^ calculated for C_29_H_25_F_3_NO_3_^+^ (*m*/*z*): 492.2, found 492.3.

*Benzyl 2-(benzyloxy)-4-((4-methoxybenzyl)amino)benzoate* (**17e**). Isolated in 75% as a pale-yellow crystalline solid. Mp: 112.7 ± 1 °C; *δ*_H_ (500 MHz, CDCl_3_-d): 3.81 (3H, s, CH_3_O), 4.25 (2H, d, *J* = 5.2 Hz, CH_2_), 4.41 (1H, t, *J* = 5.2 Hz, NH), 5.09 (2H, s, CH_2_(Bn)), 5.31 (2H, s, CH_2_(Bn)), 6.16 (1H, d, *J* = 2.1 Hz, Ar), 6.21 (1H, dd, *J* = 8.6 Hz, *J* = 2.1 Hz, Ar), 6.86–6.89 (2H, m, Ar), 7.22–7.25 (2H, m, Ar), 7.26–7.35 (6H, m, Ar), 7.38–7.41 (2H, m, Ar), 7.42–7.45 (2H, m, Ar), 7.84 (1H, d, *J* = 8.6 Hz, Ar); *δ*_C_ (126 MHz, CDCl_3_-d): 47.3, 55.4, 66.0, 70.6, 97.4, 105.1, 108.6, 114.3, 127.1, 127.7, 127.9, 128.2, 128.5, 128.6, 128.9, 130.3, 134.5, 137.0, 137.0, 153.1, 159.2, 161.1, 165.9; ESI-MS [M + H]^+^ calculated for C_29_H_28_NO_4_^+^ (*m*/*z*): 454.2, found 454.3.

#### 4.2.3. General procedure for preparation of substituted t-butyl sulphonamide acetates (19a–f)

The solution of sarcosine *t*-butyl ester hydrochloride (182 mg; 1.00 mmol) was dissolved in anhydrous CH_3_CN (3.59 ml; 3.59 ml/mmol) under N_2_ atmosphere and the mixture was cooled to 0 °C. Further, DIPEA (0.44 ml; 2.50 mmol) was added and the mixture was stirred at 0 °C for 10 min. Corresponding substituted sulphonyl chloride **18a**–**f** (1.50 mmol) was added dropwise and the mixture was stirred for 30 min at 0 °C, then it was allowed to warm to RT and stirred for 30 min. The reaction was quenched with H_2_O (5 ml) and the product was extracted to EA (5 ml). The aqueous layer was extracted with two portions of EA (both 5 ml). Organic layers were combined, washed with saturated aqueous NaHCO_3_ (8 ml), then with brine (8 ml). The organic layer was dried with Na_2_SO_4_, filtered and evaporated. The crude product was purified by glass column chromatography on silica gel in mobile phase PE:EA (20:1) to produce desired *t*-butyl esters **19a**–**f**.

*tert-Butyl 2-(2,3,4,5,6-pentafluoro-N-methylphenylsulfonamido)acetate* (**19a**). Isolated in 88% as a white crystalline solid. Mp = 86.3 ± 1 °C; *δ*_H_ (500 MHz, DMSO-d_6_): 1.38 (9H, s, *t*-Bu), 2.98 (3H, s, CH_3_N), 4.13 (2H, s, CH_2_); *δ*_C_ (126 MHz, DMSO-d_6_): 27.5, 35.2, 51.1, 82.0, 136.3–138.6 (m), 142.1–144.5 (m), 143.0–145.3 (m), 167.2; ESI-MS [M–H]^–^ calculated for C_13_H_13_F_5_NO_4_S^–^ (*m*/*z*): 374.0, found 374.2.

*tert-Butyl 2-(2,4,6-trifluoro-N-methylphenylsulfonamido)acetate* (**19b**). Isolated in 98% yield as a yellow oil. *δ*_H_ (500 MHz, DMSO-d_6_): 1.35 (9H, s, *t*-Bu), 2.95 (3H, s, CH_3_N), 4.08 (2H, s, CH_2_), 7.41–7.46 (2H, m, Ar); *δ*_C_ (126 MHz, DMSO-d_6_): 27.5, 35.0, 50.8, 81.7, 102.5 (td, *J* = 26.5 Hz, *J* = 3.6 Hz), 113.9 (td, *J* = 17.0 Hz, *J* = 5.1 Hz), 159.9 (ddd, *J* = 256.7 Hz, *J* = 16.2 Hz, *J* = 7.0 Hz), 164.5 (dt, *J* = 254.1 Hz, *J* = 16.4 Hz), 167.2; ESI-MS [M + H]^+^ calculated for C_13_H_17_F_3_NO_4_S^+^ (*m*/*z*): 340.1, found 340.1.

*tert-Butyl 2-(3,5-difluoro-N-methylphenylsulfonamido)acetate* (**19c**). Isolated in 95% as a white crystalline solid. Mp: 79.6 ± 1 °C; *δ*_H_ (500 MHz, DMSO-d_6_): 1.35 (9H, s, *t*-Bu), 2.86 (3H, s, CH_3_N), 4.01 (2H, s, CH_2_), 7.52–7.58 (2H, m, Ar), 7.65 (1H, tt, *J* = 9.2 Hz, *J* = 2.3 Hz, Ar); *δ*_C_ (126 MHz, DMSO-d_6_): 27.5, 35.5, 51.0, 81.5, 108.6 (t, *J* = 25.8 Hz), 110.7–111.0 (m), 141.6 (t, *J* = 8.6 Hz), 162.3 (dd, *J* = 251.3 Hz, *J* = 12.4 Hz), 167.3; ESI-MS [M + H]^+^ calculated for C_13_H_18_F_2_NO_4_S^+^ (*m*/*z*): 322.1, found 322.1.

*tert-Butyl 2-(4-fluoro-N-methylphenylsulfonamido)acetate* (**19d**). Isolated in 95% as a white crystalline solid. Mp: 94.5 ± 1 °C; *δ*_H_ (500 MHz, DMSO-d_6_): 1.34 (9H, s, *t*-Bu), 2.80 (3H, s, CH_3_N), 3.91 (2H, s, CH_2_), 7.42–7.47 (2H, m, Ar), 7.84–7.89 (2H, m, Ar); *δ*_C_ (126 MHz, DMSO-d_6_): 27.5, 35.4, 51.1, 81.4, 116.4 (d, *J* = 22.7 Hz), 130.1 (d, *J* = 9.6 Hz), 134.6 (d, *J* = 2.9 Hz), 164.4 (d, *J* = 251.4 Hz), 167.4; ESI-MS [M + H]^+^ calculated for C_13_H_19_FNO_4_S^+^ (*m*/*z*): 304.1, found 304.1.

*tert-Butyl 2-(N-methyl-N-(p-toluenesulfonamido)acetate* (**19e**). Isolated in 98% yield as a yellow oil. *δ*_H_ (500 MHz, CDCl_3_-d): 1.30 (9H, s, *t*-Bu), 2.31 (3H, s, CH_3_(tol)), 2.77 (3H, s, CH_3_N), 3.75 (2H, s, CH_2_), 7.19–7.23 (2H, m, Ar), 7.56–7.61 (2H, m, Ar); *δ*_C_ (126 MHz, CHCl_3_-d): 21.3, 27.7, 35.4, 51.4, 81.8, 127.2, 129.5, 135.3, 143.3, 167.3; ESI-MS [M + H]^+^ calculated for C_14_H_22_NO_4_S^+^ (*m*/*z*): 300.1, found 300.2.

*tert-Butyl 2-(N-methylphenylsulfonamido)acetate* (**19f**). Isolated in 98% yield as a white crystalline solid. Mp: 60.9 ± 1 °C; *δ*_H_ (500 MHz, DMSO-d_6_): 1.34 (9H, s, *t*-Bu), 2.81 (3H, s, CH_3_N), 3.90 (2H, s, CH_2_), 7.58–7.63 (2H, m, Ar), 7.66–7.70 (1H, m, Ar), 7.77–7.81 (2H, m, Ar); *δ*_C_ (126 MHz, DMSO-d_6_): 27.5, 35.4, 51.1, 81.3, 126.9, 129.3, 132.8, 138.1, 167.4; ESI-MS [M + H]^+^ calculated for C_13_H_20_NO_4_S^+^ (*m*/*z*): 286.1, found 286.1.

#### 4.2.4. General procedure for preparation of substituted carboxylic acid (20a–f)

To a solution of substituted *t*-butyl ester sulphonamide acetates **19a**–**f** (1 mmol) in CH_2_Cl_2_ (6.3 ml; 6.3 ml/mmol), TFA (6.3 ml; 6.3 ml/mmol) was added and the mixture was stirred at RT for 3.5 h. Solvent and TFA were then evaporated under reduced pressure, to give pure desired substituted acids **20a**–**f**.

*2-(2,3,4,5,6-Pentafluoro-N-methylphenylsulfonamido)acetic acid* (**20a**). Isolated in quantitative yield as a white crystalline solid. Mp: 192.0 ± 1 °C; *δ*_H_ (500 MHz, DMSO-d_6_): 2.97 (3H, s, CH_3_N), 4.12 (2H, s, CH_2_), 13.08 (1H, bs, COOH); *δ*_C_ (126 MHz, DMSO-d_6_): 35.2, 50.5, 136.3–138.7 (m), 142.0–144.4 (m), 143.1–145.4 (m), 169.5; ESI-MS [M–H]^–^ calculated for C_9_H_5_F_5_NO_4_S^–^ (*m*/*z*): 318.0, found 318.0.

*2-(2,4,6-Trifluoro-N-methylphenylsulfonamido)acetic acid* (**20b**). Isolated in 94% as a white crystalline solid. Mp: 166.7 ± 1 °C; *δ*_H_ (500 MHz, DMSO-d_6_): 2.94 (3H, s, CH_3_N), 4.07 (2H, s, CH_2_), 7.40–7.46 (2H, m, Ar), 12.93 (1H, bs, COOH); *δ*_C_ (126 MHz, DMSO-d_6_): 35.1, 50.3, 102.5 (td, *J* = 28.8 Hz, *J* = 3.7 Hz), 113.9 (td, *J* = 17.5 Hz, *J* = 5.4 Hz), 159.9 (ddd, *J* = 256.6 Hz, *J* = 16.2 Hz, *J* = 7.1 Hz), 164.5 (dt, *J* = 254.1 Hz, *J* = 16.6 Hz), 169.6; ESI-MS [M–H]^–^ calculated for C_9_H_7_F_3_NO_4_S^–^ (*m*/*z*): 282.0, found 282.1.

*2-(3,5-Difluoro-N-methylphenylsulfonamido)acetic acid* (**20c**). Isolated in 95% as a grey crystalline solid. Mp: 133.1 ± 1 °C; *δ*_H_ (500 MHz, DMSO-d_6_): 2.84 (3H, s, CH_3_N), 3.99 (2H, s, CH_2_), 7.53–7.58 (2H, m, Ar), 7.63 (1H, tt, *J* = 9.2 Hz, *J* = 2.3 Hz, Ar), 12.89 (1H, bs, COOH); *δ*_C_ (126 MHz, DMSO-d_6_): 35.5, 50.5, 108.6 (t, *J* = 25.8 Hz), 110.8–111.0 (m), 141.6 (t, *J* = 8.5 Hz), 162.3 (dd, *J* = 251.2 Hz, *J* = 12.4 Hz), 169.8; ESI-MS [M–H]^–^ calculated for C_9_H_8_F_2_NO_4_S^–^ (*m*/*z*): 264.0, found 264.1.

*2-(4-Fluoro-N-methylphenylsulfonamido)acetic acid* (**20d**). Isolated in 99% yield as a white crystalline solid. Mp: 161.2 ± 1 °C; *δ*_H_ (500 MHz, DMSO-d_6_): 2.79 (3H, s, CH_3_N), 3.91 (2H, s, CH_2_), 7.41–7.46 (2H, m, Ar), 7.84–7.89 (2H, m, Ar), 12.82 (1H, m, COOH); *δ*_C_ (126 MHz, DMSO-d_6_): 35.5, 50.5, 116.4 (d, *J* = 22.6 Hz), 130.1 (d, *J* = 9.6 Hz), 134.5 (d, *J* = 2.9 Hz), 164.5 (d, *J* = 251.3 Hz), 169.8; ESI-MS [M–H]^–^ calculated for C_9_H_9_FNO_4_S^–^ (*m*/*z*): 246.0, found 246.1.

*2-(N-Methyl-N-(p-toluenesulfonamido)acetic acid* (**20e**). Isolated in 99% yield as a brown crystalline solid. Mp: 149.0 ± 1 °C; *δ*_H_ (500 MHz, DMSO-d_6_): 2.38 (3H, s, CH_3_(tol)), 2.75 (3H, s, CH_3_N), 3.85 (2H, s, CH_2_), 7.38–7.42 (2H, m, Ar), 7.66–7.69 (2H, m, Ar), 12.71 (1H, bs, COOH); *δ*_C_ (126 MHz, DMSO-d_6_): 21.0, 35.5, 50.6, 127.1, 129.8, 135.0, 143.3, 169.9; ESI-MS [M–H]^–^ calculated for C_10_H_12_NO_4_S^–^ (*m*/*z*): 242.1, found 242.1.

*2-(N-Methylphenylsulfonamido)acetic acid* (**20f**). Isolated in 97% yield as a grey crystalline solid. Mp: 181.1 ± 1 °C; *δ*_H_ (500 MHz, DMSO-d_6_): 2.78 (3H, s, CH_3_N), 3.89 (2H, s, CH_2_), 7.58–7.63 (2H, m, Ar), 7.65–7.69 (1H, m, Ar), 7.78–7.81 (2H, m, Ar), 12.47 (1H, bs, COOH); *δ*_C_ (126 MHz, DMSO-d_6_): 35.6, 50.6, 127.0, 129.4, 132.9, 138.0, 169.9; ESI-MS [M–H]^–^ calculated for C_9_H_10_NO_4_S^–^ (*m*/*z*): 228.0, found 228.1.

#### 4.2.5. General procedure for the key amide coupling reaction (21a–n)

Selected acid **20a**–**f** (0.8 mmol) and Ph_3_PCl_2_ (666 mg; 2.00 mmol) were dissolved under N_2_ atmosphere in anhydrous CHCl_3_ (3.57 ml; 7.13 ml/mmol) in a 35 ml microwave pressure vessel. The reaction mixture was stirred for 1 h at RT, then the selected secondary amine **17a**–**e** (0.5 mmol) was quantitatively transferred to the reaction in anhydrous CHCl_3_ (3.57 ml; 7.13 ml/mmol). The pressure vial was transferred to a microwave reactor and heated to 65 °C for 1.5 h. The mixture was transferred to a separatory funnel, then saturated aqueous NaHCO_3_ (4 ml) and H_2_O (4 ml) were added. After extraction and separation, the aqueous phase was extracted with two portions of CHCl_3_ (both 5 ml). Organic phases were combined, washed with saturated aqueous NaHCO_3_ (7 ml) and brine (7 ml), dried with Na_2_SO_4_, filtered and evaporated. The residue was purified by column glass chromatography on silica gel in mobile phase heptane:EA (3:1) to produce desired debenzylated amides **21a**–**n**.

*Benzyl 2-(benzyloxy)-4-(N-(4-cyclohexylbenzyl)-2-(2,3,4,5,6-pentafluoro-N-methylphenylsulfonamido)acetamido)benzoate* (**21a**). Isolated in 58% yield as a white crystalline solid. Mp: 56.5 ± 1 °C; *δ*_H_ (500 MHz, CDCl_3_-d): 1.19–1.29 (1H, m, Cy), 1.32–1.43 (4H, m, Cy), 1.71–1.77 (1H, m, Cy), 1.78–1.87 (4H, m, Cy), 2.43–2.50 (1H, m, Cy), 3.05 (3H, s, CH_3_N), 3.85 (2H, s, CH_2_), 4.67 (2H, s, CH_2_), 4.94 (2H, s, CH_2_(Bn)), 5.35 (2H, s, CH_2_(Bn)), 6.42–6.45 (1H, m, Ar), 6.65 (1H, dd, *J* = 8.2 Hz, *J* = 1.6 Hz, Ar), 6.94 (2H, d, *J* = 8.0 Hz, Ar), 7.11 (2H, d, *J* = 8.0 Hz, Ar), 7.28–7.36 (8H, m, Ar), 7.37–7.40 (2H, m, Ar), 7.83 (1H, d, *J* = 8.2 Hz, Ar); *δ*_C_ (126 MHz, CDCl_3_-d): 26.2, 27.0, 34.6, 36.1, 44.4, 52.2, 53.0, 67.2, 70.9, 114.2, 120.1, 121.4, 127.2, 127.3, 128.3, 128.4, 128.4, 128.7, 128.8, 129.0, 133.5, 133.6, 135.8, 135.8, 136.6–139.1 (m), 143.8–146.1 (m), 144.4, 148.1, 158.9, 165.4, 166.0; ESI-MS [M + H]^+^ calculated for C_43_H_40_F_5_N_2_O_6_S^+^ (*m*/*z*): 807.2, found 807.4.

*Benzyl 2-(benzyloxy)-4-(N-(4-cyclohexylbenzyl)-2-(N-methyl-N-(p-toluenesulfonyl))acetamido)benzoate* (**21b**). Isolated in 28% as a yellow oil. *δ*_H_ (500 MHz, CDCl_3_-d): 1.23–1.26 (1H, m, Cy), 1.33–1.38 (4H, m, Cy), 1.70–1.74 (1H, m, Cy), 1.78–1.84 (4H, m, Cy), 2.39 (3H, s, CH_3_(tol.)), 2.42–2.48 (1H, m, Cy), 2.80 (3H, s, CH_3_N), 3.64 (2H, s, CH_2_), 4.74 (2H, s, CH_2_), 4.93 (2H, s, CH_2_(Bn)), 5.34 (2H, s, CH_2_(Bn)), 6.48–6.51 (1H, m, Ar), 6.66 (1H, dd, *J* = 8.2 Hz, *J* = 1.2 Hz, Ar), 7.00 (2H, d, *J* = 8.0 Hz, Ar), 7.09 (2H, d, *J* = 8.0 Hz, Ar), 7.23–7.25 (2H, m, Ar), 7.27–7.34 (8H, m, Ar), 7.37–7.39 (2H, m, Ar), 7.60 (2H, d, *J* = 8.2 Hz, Ar), 7.82 (1H, d, *J* = 8.2 Hz, Ar); *δ*_C_ (126 MHz, CDCl_3_-d): 21.7, 26.2, 27.0, 34.6, 36.1, 44.4, 51.5, 53.0, 67.2, 70.9, 127.1, 127.3, 127.7, 128.2, 128.4, 128.4, 128.7, 128.8, 129.1, 129.6, 133.3, 134.1, 135.9, 136.0, 143.5, 147.9, 158.9, 165.6, 166.9; ESI-MS [M + H]^+^ calculated for C_44_H_47_N_2_O_6_S^+^ (*m*/*z*): 731.3, found 731.5.

*Benzyl 2-(benzyloxy)-4-(N-(4-tert-butylbenzyl)-2-(2,3,4,5,6-pentafluoro-N-methylphenylsulfonamido)acetamido)benzoate* (**21c**). Isolated in 68% as a white crystalline solid. Mp: 188.3 ± 1 °C; *δ*_H_ (500 MHz, CDCl_3_-d): 1.30 (9H, s, *t*-Bu), 3.05 (3H, s, CH_3_N), 3.86 (2H, s, CH_2_), 4.68 (2H, s, CH_2_), 4.95 (2H, s, CH_2_(Bn)), 5.35 (2H, s, CH_2_(Bn)), 6.47 (1H, s, Ar), 6.66 (1H, dd, *J* = 8.2 Hz, *J* = 1.5 Hz, Ar), 6.96 (2H, d, *J* = 8.1 Hz, Ar), 7.28–7.36 (10H, m, Ar), 7.37–7.40 (2H, m, Ar), 7.84 (1H, d, *J* = 8.1 Hz, Ar); *δ*_C_ (126 MHz, CDCl_3_-d): 31.5, 34.7, 36.1, 52.2, 52.9, 67.3, 70.9, 114.2, 120.1, 121.4, 125.7, 127.3, 128.3, 128.4, 128.4, 128.7, 128.7, 128.8, 133.2, 133.5, 135.8, 135.8, 136.7–139.1 (m), 143.8–146.1 (m), 144.5, 151.2, 158.9, 165.4, 166.0; ESI-MS [M + H]^+^ calculated for C_41_H_38_F_5_N_2_O_6_S^+^ (*m*/*z*): 781.2, found 781.1.

*Benzyl 2-(benzyloxy)-4-(N-(4-tert-butylbenzyl)-2-(N-methyl-N-(p-toluenesulfonyl))acetamido)benzoate* (**21d**). Isolated in 31% yield as a yellow oil. *δ*_H_ (500 MHz, CDCl_3_-d): 1.29 (9H, s, *t*-Bu), 2.40 (3H, s, CH_3_(tol)), 2.82 (3H, s, CH_3_N), 3.66 (2H, s, CH_2_), 4.76 (2H, s, CH_2_), 4.96 (2H, s, CH_2_(Bn)), 5.35 (2H, s, CH_2_(Bn)), 6.52–6.57 (1H, m, Ar), 6.69 (1H, dd, *J* = 8.2 Hz, *J* = 1.5 Hz, Ar), 7.04 (2H, d, *J* = 8.2 Hz, Ar), 7.24–7.27 (2H, m, Ar), 7.27–7.35 (10H, m, Ar), 7.38–7.41 (2H, m, Ar), 7.61 (2H, d, *J* = 8.2 Hz, Ar), 7.84 (1H, d, *J* = 8.2 Hz, Ar); *δ*_C_ (126 MHz, CDCl_3_-d): 21.7, 31.5, 34.7, 36.1, 51.5, 52.9, 67.2, 70.9, 114.4, 120.2, 120.8, 125.6, 125.8, 127.2, 127.6, 128.2, 128.4, 128.4, 128.7, 128.7, 129.6, 133.3, 133.7, 135.5, 135.9, 135.9, 143.4, 145.3, 150.9, 158.9, 165.5, 166.9; ESI-MS [M + H]^+^ calculated for C_42_H_45_N_2_O_6_S^+^ (*m*/*z*): 705.3, found 705.4.

*Benzyl 2-(benzyloxy)-4-(N-(benzyl)-2-(2,3,4,5,6-pentafluoro-N-methylphenylsulfonamido)acetamido)benzoate* (**21e**). Isolated in 32% yield as a yellow oil. *δ*_H_ (500 MHz, CDCl_3_-d): 3.05 (3H, s, CH_3_N), 3.87 (2H, s, CH_2_), 4.71 (2H, s, CH_2_), 4.98 (2H, s, CH_2_(Bn)), 5.35 (2H, s, CH_2_(Bn)), 6.47–6.51 (1H, m, Ar), 6.62 (1H, dd, *J* = 8.2 Hz, *J* = 1.4 Hz, Ar), 7.00–7.04 (2H, m, Ar), 7.25–7.29 (3H, m, Ar), 7.29–7.36 (8H, m, Ar), 7.37–7.40 (2H, m, Ar), 7.82 (1H, d, *J* = 8.2 Hz, Ar); *δ*_C_ (126 MHz, CDCl_3_-d): 36.1, 52.2, 53.2, 67.3, 70.9, 114.1, 120.1, 121.5, 127.2, 128.1, 128.3, 128.4, 128.7, 128.8, 128.8, 128.9, 133.5, 135.8, 135.8, 136.2, 136.6–139.0 (m), 143.7–146.1 (m), 144.3, 159.0, 165.4, 166.2; ESI-MS [M + H]^+^ calculated for C_37_H_30_F_5_N_2_O_6_S^+^ (*m*/*z*): 725.2, found 725.3.

*Benzyl 2-(benzyloxy)-4-(N-(benzyl)-2-(N-methyl-N-(p-toluenesulfonyl))acetamido)benzoate* (**21f**). Isolated in 19% yield as a yellow oil. *δ*_H_ (500 MHz, CDCl_3_-d): 2.40 (3H, s, CH_3_(tol)), 2.82 (3H, s, CH_3_N), 3.67 (2H, s, CH_2_), 4.79 (2H, s, CH_2_), 4.99 (2H, s, CH_2_(Bn)), 5.35 (2H, s, CH_2_(Bn)), 6.55–6.58 (1H, m, Ar), 6.65 (1H, dd, *J* = 8.2 Hz, *J* = 1.2 Hz, Ar), 7.07–7.12 (2H, m, Ar), 7.24–7.28 (5H, m, Ar), 7.28–7.36 (8H, m, Ar), 7.38–7.41 (2H, m, Ar), 7.60–7.62 (2H, m, Ar), 7.82 (1H, d, *J* = 8.2 Hz, Ar); *δ*_C_ (126 MHz, CDCl_3_-d): 21.7, 36.1, 51.5, 53.2, 67.2, 70.9, 114.3, 120.2, 127.2, 127.6, 127.9, 128.2, 128.4, 128.4, 128.7, 128.8, 129.0, 129.6, 133.3, 135.5, 135.9, 136.0, 136.7, 143.5, 145.1, 158.9, 165.5, 167.0; ESI-MS [M + H]^+^ calculated for C_38_H_37_N_2_O_6_S^+^ (*m*/*z*): 649.2, found 649.3.

*Benzyl 2-(benzyloxy)-4-(N-(4-trifluoromethylbenzyl)-2-(2,3,4,5,6-pentafluoro-N-methylphenylsulfonamido)acetamido)benzoate* (**21g**). Isolated in 66% yield as a yellow oil. *δ*_H_ (500 MHz, CDCl_3_-d): 3.05 (3H, s, CH_3_N), 3.90 (2H, s, CH_2_), 4.74 (2H, s, CH_2_), 5.05 (2H, s, CH_2_(Bn)), 5.36 (2H, s, CH_2_(Bn)), 6.54 (1H, d, *J* = 1.7 Hz, Ar), 6.63 (1H, dd, *J* = 8.2 Hz, *J* = 1.7 Hz, Ar), 7.15 (2H, d, *J* = 8.0 Hz, Ar), 7.31–7.36 (8H, m, Ar), 7.37–7.41 (2H, m, Ar), 7.52 (2H, d, *J* = 8.0 Hz, Ar), 7.84 (1H, d, *J* = 8.0 Hz, Ar); *δ*_C_ (126 MHz, CDCl_3_-d): 36.1, 52.1, 52.9, 67.3, 71.0, 113.8, 119.9, 121.8, 125.8 (q, *J* = 3.8 Hz), 127.1, 128.4, 128.5, 128.5, 128.7, 128.9, 129.1, 130.4 (q, *J* = 32.5 Hz), 133.7, 135.6, 135.7, 136.7–139.1 (m), 140.1, 143.7–146.0 (m), 144.1, 159.1, 165.3, 166.5; ESI-MS [M + H]^+^ calculated for C_38_H_29_F_8_N_2_O_6_S^+^ (*m*/*z*): 793.2, found 793.3.

*Benzyl 2-(benzyloxy)-4-(N-(4-trifluoromethylbenzyl)-2-(2,4,6-trifluoro-N-methylphenylsulfonamido)acetamido)benzoate* (**21h**). Isolated in 18% yield as a yellow oil. *δ*_H_ (500 MHz, CDCl_3_-d): 3.05 (3H, s, CH_3_N), 3.87 (2H, s, CH_2_), 4.77 (2H, s, CH_2_), 5.05 (2H, s, CH_2_(Bn)), 5.35 (2H, s, CH_2_(Bn)), 6.58 (1H, d, *J* = 1.5 Hz, Ar), 6.65 (1H, dd, *J* = 8.2 Hz, *J* = 1.5 Hz, Ar), 6.67–6.72 (2H, m, Ar), 7.18 (2H, d, *J* = 8.1 Hz, Ar), 7.30–7.36 (8H, m, Ar), 7.38–7.41 (2H, m, Ar), 7.51 (2H, d, *J* = 8.1 Hz, Ar), 7.81 (1H, d, *J* = 8.2 Hz, Ar); *δ*_C_ (126 MHz, CDCl_3_-d): 36.1, 51.7, 52.9, 67.3, 71.0, 101.7–102.2 (m), 113.9, 119.9, 121.6, 124.1 (q, *J* = 271.9 Hz), 125.7 (q, *J* = 3.7 Hz), 127.1, 128.3, 128.5, 128.7, 128.8, 129.2, 130.2 (q, *J* = 32.5 Hz), 133.6, 135.8, 135.8, 140.5, 144.6, 159.1, 160.8 (ddd, *J* = 259.0 Hz, *J* = 15.4 Hz, *J* = 7.1 Hz), 164.9 (dt, *J* = 31.7 Hz, *J* = 15.6 Hz), 165.4, 166.9; ESI-MS [M + H]^+^ calculated for C_38_H_31_F_6_N_2_O_6_S^+^ (*m*/*z*): 757.2, found 757.1.

*Benzyl 2-(benzyloxy)-4-(N-(4-trifluoromethylbenzyl)-2-(3,5-difluoro-N-methylphenylsulfonamido)acetamido)benzoate* (**21i**). Isolated in 21% yield as a yellow oil. *δ*_H_ (500 MHz, CDCl_3_-d): 2.89 (3H, s, CH_3_N), 3.72 (2H, s, CH_2_), 4.79 (2H, s, CH_2_), 5.05 (2H, s, CH_2_(Bn)), 5.36 (2H, s, CH_2_(Bn)), 6.53–6.55 (1H, m, Ar), 6.63 (1H, dd, *J* = 8.2 Hz, *J* = 1.7 Hz, Ar), 7.02 (1H, tt, *J* = 8.5 Hz, *J* = 2.3 Hz, Ar), 7.19 (2H, d, *J* = 8.1 Hz, Ar), 7.28–7.36 (10H, m, Ar), 7.38–7.41 (2H, m, Ar), 7.53 (2H, d, *J* = 8.1 Hz, Ar), 7.84 (1H, d, *J* = 8.2 Hz, Ar); *δ*_C_ (126 MHz, CDCl_3_-d): 36.3, 51.6, 52.9, 67.3, 71.0, 108.4 (t, *J* = 25.1 Hz), 111.0–111.3 (m), 113.9, 119.9, 121.6, 124.1 (q, *J* = 272.0 Hz), 125.8 (q, *J* = 3.6 Hz), 127.1, 128.3, 128.4, 128.7, 128.9, 129.2, 130.3 (q, *J* = 32.5 Hz), 133.6, 135.7, 135.8, 140.5, 142.3 (t, *J* = 8.4 Hz), 144.5, 159.1, 162.8 (dd, *J* = 254.0 Hz, *J* = 11.6 Hz), 165.3, 166.9, 171.3; ESI-MS [M + H]^+^ calculated for C_38_H_32_F_5_N_2_O_6_S^+^ (*m*/*z*): 739.2, found 739.2.

*Benzyl 2-(benzyloxy)-4-(N-(4-trifluoromethylbenzyl)-2-(4-fluoro-N-methylphenylsulfonamido)acetamido)benzoate* (**21j**). Isolated in 21% yield as a yellow oil. *δ*_H_ (500 MHz, CDCl_3_-d): 2.83 (3H, s, CH_3_N), 3.70 (2H, s, CH_2_), 4.81 (2H, s, CH_2_), 5.05 (2H, s, CH_2_(Bn)), 5.36 (2H, s, CH_2_(Bn)), 6.58–6.60 (1H, m, Ar), 6.65 (1H, d, *J* = 8.1 Hz, *J* = 1.7 Hz, Ar), 7.11–7.15 (2H, m, Ar), 7.21 (2H, d, *J* = 8.2 Hz, Ar), 7.28–7.36 (10H, m, Ar), 7.38–7.42 (2H, m, Ar), 7.52 (2H, d, *J* = 8.2 Hz, Ar), 7.84 (1H, d, *J* = 8.1 Hz, Ar); *δ*_C_ (126 MHz, CDCl_3_-d): 36.2, 51.5, 53.0, 67.3, 71.0, 114.0, 116.2 (d, *J* = 22.5 Hz), 125.7 (q, *J* = 3.6 Hz), 127.1, 127.4, 128.3, 128.5, 128.6, 128.7, 128.8, 129.2, 130.3 (d, *J* = 9.3 Hz), 133.5, 135.8, 140.6, 142.1, 144.8, 165.2 (d, *J* = 254.7 Hz), 165.4, 165.9, 167.3; ESI-MS [M + H]^+^ calculated for C_38_H_33_F_4_N_2_O_6_S^+^ (*m*/*z*): 721.2, found 721.2.

*Benzyl 2-(benzyloxy)-4-(N-(4-trifluoromethylbenzyl)-2-(N-methyl-N-(p-toluenesulfonyl))acetamido)benzoate* (**21k**). Isolated in 50% yield as a yellow oil. *δ*_H_ (500 MHz, CDCl_3_-d): 2.40 (3H, s, CH_3_(tol)), 2.82 (3H, s, CH_3_N), 3.67 (2H, s, CH_2_), 4.83 (2H, s, CH_2_), 5.06 (2H, s, CH_2_(Bn)), 5.36 (2H, s, CH_2_(Bn)), 6.61–6.62 (1H, m, Ar), 6.65 (1H, dd, *J* = 8.2 Hz, *J* = 1.8 Hz, Ar), 7.22 (2H, d, *J* = 8.1 Hz, Ar), 7.25 (2H, d, *J* = 8.3 Hz, Ar), 7.28–7.36 (8H, m, Ar), 7.38–7.42 (2H, m, Ar), 7.52 (2H, d, *J* = 8.1 Hz, Ar), 7.60 (2H, d, *J* = 8.3 Hz, Ar), 7.84 (1H, d, *J* = 8.2 Hz, Ar); *δ*_C_ (126 MHz, CDCl_3_-d): 21.6, 36.3, 51.5, 52.9, 67.2, 70.9, 114.1, 119.9, 124.1 (q, *J* = 271.4 Hz), 125.6 (q, *J* = 3.6 Hz), 127.1, 127.6, 128.2, 128.4, 128.7, 128.8, 129.2, 129.7, 130.1 (q, *J* = 32.6 Hz), 133.4, 135.8, 140.7, 143.6, 144.9, 159.0, 165.4, 167.4; ESI-MS [M + H]^+^ calculated for C_39_H_36_F_3_N_2_O_6_S^+^ (*m*/*z*): 717.2, found 717.3.

*Benzyl 2-(benzyloxy)-4-(N-(4-trifluoromethylbenzyl)-2-(N-methylphenylsulfonyl))acetamido)benzoate* (**21l**). Isolated in 38% yield as a pale-yellow oil. *δ*_H_ (500 MHz, CDCl_3_-d): 2.84 (3H, s, CH_3_N), 3.68 (2H, s, CH_2_), 4.82 (2H, s, CH_2_), 5.06 (2H, s, CH_2_(Bn)), 5.36 (2H, s, CH_2_(Bn)), 6.59–6.61 (1H, m, Ar), 6.63–6.66 (1H, m, Ar), 7.22 (2H, d, *J* = 8.0 Hz, Ar), 7.27–7.36 (8H, m, Ar), 7.38–7.42 (2H, m, Ar), 7.44–7.48 (2H, m, Ar), 7.51 (2H, d, *J* = 8.0 Hz, Ar), 7.53–7.57 (1H, m, Ar), 7.71–7.73 (2H, m, Ar), 7.84 (1H, dd, *J* = 8.2 Hz, *J* = 1.0 Hz, Ar); *δ*_C_ (126 MHz, CDCl_3_-d): 35.3, 50.5, 51.9, 66.3, 70.0, 113.1, 119.0, 120.4, 123.1 (q, *J* = 272.0 Hz), 124.7 (q, *J* = 3.7 Hz), 126.1, 126.5, 127.2, 127.4, 127.5, 127.7, 127.8, 128.1, 128.2, 129.2 (q, *J* = 32.5 Hz), 131.8, 132.5, 134.8, 137.5, 139.7, 143.9, 158.0, 164.4, 166.3; ESI-MS [M + H]^+^ calculated for C_38_H_34_F_3_N_2_O_6_S^+^ (*m*/*z*): 703.2, found 703.3.

*Benzyl 2-(benzyloxy)-4-(N-(4-methoxybenzyl)-2-(2,4,6-trifluoro-N-methylphenylsulfonamido)acetamido)benzoate* (**21m**). Isolated in 51% as a yellow oil. *δ*_H_ (500 MHz, CDCl_3_-d): 3.05 (3H, s, CH_3_N), 3.78 (3H, s, CH_3_O), 3.83 (2H, s, CH_2_), 4.66 (2H, s, CH_2_), 5.02 (2H, s, CH_2_(Bn)), 5.35 (2H, s, CH_2_(Bn)), 6.53–6.55 (1H, m, Ar), 6.62 (1H, dd, *J* = 8.2 Hz, *J* = 1.7 Hz, Ar), 6.69–6.75 (2H, m, Ar), 6.75–6.79 (2H, m, Ar), 6.93–6.97 (2H, m, Ar), 7.28–7.37 (8H, m, Ar), 7.38–7.40 (2H, m, Ar), 7.82 (1H, d, *J* = 8.2 Hz, Ar); *δ*_C_ (126 MHz, CDCl_3_-d): 36.1, 51.8, 52.6, 55.4, 67.2, 71.0, 101.7–102.2 (m), 114.0, 120.3, 127.2, 128.2, 128.4, 128.4, 128.6, 128.7, 128.8, 130.4, 133.4, 135.9, 135.9, 144.8, 159.0, 159.4, 165.5, 166.4; ESI-MS [M + H]^+^ calculated for C_38_H_34_F_3_N_2_O_7_S^+^ (*m*/*z*): 719.2, found 719.3.

*Benzyl 2-(benzyloxy)-4-(N-(4-methoxybenzyl)-2-(2,3,4,5,6-pentafluoro-N-methylphenylsulfonamido)acetamido)benzoate* (**21n**). Isolated in 42% yield as a yellow oil. *δ*_H_ (500 MHz, CDCl_3_-d): 3.05 (3H, s, CH_3_N), 3.78 (3H, s, CH_3_O), 3.85 (2H, s, CH_2_), 4.64 (2H, s, CH_2_), 5.02 (2H, s, CH_2_(Bn)), 5.35 (2H, s, CH_2_(Bn)), 6.48–6.51 (1H, m, Ar), 6.59 (1H, dd, *J* = 8.1 Hz, *J* = 1.2 Hz, Ar), 6.78 (2H, d, *J* = 8.5 Hz, Ar), 6.93 (2H, d, *J* = 8.5 Hz, Ar), 7.29–7.37 (8H, m, Ar), 7.38–7.41 (2H, m, Ar), 7.82 (1H, d, *J* = 8.1 Hz, Ar); *δ*_C_ (126 MHz, CDCl_3_-d): 36.2, 52.2, 52.6, 55.4, 67.3, 71.0, 114.1, 114.2, 120.2, 127.2, 128.3, 128.4, 128.4, 128.7, 128.8, 130.3, 133.5, 135.8, 144.3, 159.0, 159.5, 165.4, 166.0; ESI-MS [M + H]^+^ calculated for C_38_H_32_F_5_N_2_O_7_S^+^ (*m*/*z*): 755.2, found 755.3.

#### 4.2.6. General procedure for preparation of final products (1–14)

To a stirring mixture of 5% Pd/C (200 mg; 10 mg/mmol) in dry MeOH (16 ml; 16 ml/mmol) in a two-neck round bottom flask connected to the Schlenk apparatus, dibenzylated amide **21a**–**n** (1 mmol) in dry THF (16 ml; 16 ml/mmol) was quantitatively transferred under N_2_ atmosphere. The mixture was evacuated, and then H_2_ was introduced into the flask. The mixture was stirred for 3 h at RT, then filtered through a column of celite on a glass frit and washed with MeOH. The solvent was evaporated under reduced pressure, and the residue was purified by glass column chromatography on silica gel in a mobile phase CH_2_Cl_2_:MeOH:AcOH (150:1:1) to give final *p*-aminosalicylic acids **1**–**14**.

*4-(N-(4-Cyclohexylbenzyl)-2-(2,3,4,5,6-pentafluoro-N-methylphenylsulfonamido)acetamido)-2-hydroxybenzoic acid* (**1**). Isolated in 96% yield as a white solid. Mp: 89.0 ± 1 °C; *δ*_H_ (500 MHz, CD_3_OD-d_4_): 1.23–1.32 (1H, m, Cy), 1.36–1.47 (4H, m, Cy), 1.71–1.77 (1H, m, Cy), 1.78–1.86 (4H, m, Cy), 2.44–2.50 (1H, m, Cy), 3.07 (3H, s, CH_3_N), 4.10 (2H, s, CH_2_), 4.77 (2H, s, CH_2_), 6.62 (1H, dd, *J* = 8.4 Hz, *J* = 2.0 Hz, Ar), 6.69 (1H, d, *J* = 2.0 H, Ar), 7.02 (2H, d, *J* = 8.0 Hz, Ar), 7.12 (2H, d, *J* = 8.1 Hz, Ar), 7.87 (1H, d, *J* = 8.4 Hz, Ar); *δ*_C_ (126 MHz, CD_3_OD-d_4_): 27.2, 28.0, 35.6, 36.4, 45.7, 52.9, 53.8, 114.1–114.5 (m), 117.9, 119.9, 128.0, 129.5, 133.1, 135.1, 138.0–140.5 (m), 143.9–146.3 (m), 145.0–147.3 (m), 147.7, 148.9, 164.0, 168.1, 172.6; HRMS (HESI^+^): [M + H]^+^: calculated for C_29_H_28_F_5_N_2_O_6_S^+^ (*m*/*z*): 627.1588, found 627.1585.

*4-(N-(4-Cyclohexylbenzyl)-2-(N-methyl-N-(p-toluenesulfonyl))acetamido)-2-hydroxybenzoic acid* (**2**). Isolated in 63% yield as a pale-pink crystalline solid. Mp: 125 ± 1 °C; *δ*_H_ (500 MHz, CD_3_OD-d_4_): 1.27–1.31 (1H, m, Cy), 1.37–1.43 (4H, m, Cy), 1.71–1.75 (1H, m, Cy), 1.78–1.84 (4H, m, Cy), 2.37 (3H, s, CH_3_(tol.)), 2.43–2.48 (1H, m, Cy), 2.82 (3H, s, CH_3_N), 3.74 (2H, s, CH_2_), 4.80 (2H, s, CH_2_), 6.53 (1H, d, *J* = 6.4 Hz, Ar), 6.60 (1H, s, Ar), 7.06–7.12 (4H, m, Ar), 7.29 (2H, d, *J* = 8.0 Hz, Ar), 7.55 (2H, d, *J* = 8.1 Hz, Ar), 7.86 (1H, d, *J* = 6.6 Hz, Ar); *δ*_C_ (126 MHz, CD_3_OD-d_4_): 21.5, 27.2, 28.0, 35.6, 36.9, 45.6, 52.5, 53.8, 117.2, 119.1, 127.9, 128.5, 129.6, 130.7, 133.1, 135.4, 136.3, 145.0, 146.4, 148.7, 163.6, 169.2; ESI-MS [M + H]^+^ calculated for C_30_H_35_N_2_O_6_S^+^ (*m*/*z*): 551.2, found 551.3.

*4-(N-(Benzyl)-2-(N-methyl-N-(p-toluenesulfonyl))acetamido)-2-hydroxybenzoic acid* (**3**). Isolated in 65% yield as a pale-yellow crystalline solid. Mp: 131.0 ± 1 °C; *δ*_H_ (500 MHz, DMSO-d_6_): 2.36 (3H, s, CH_3_(tol)), 2.80 (3H, s, CH_3_N), 3.82 (2H, s, CH_2_), 4.77 (2H, s, CH_2_), 6.44–6.46 (1H, m, Ar), 6.51 (1H, d, *J* = 1.9 Hz, Ar), 7.15 (2H, d, *J* = 7.1 Hz, Ar), 7.21–7.25 (1H, m, Ar), 7.26–7.31 (2H, m, Ar), 7.35 (2H, d, *J* = 8.2 Hz, Ar), 7.54 (2H, d, *J* = 8.3 Hz, Ar), 7.67 (1H, d, *J* = 8.1 Hz, Ar); *δ*_C_ (126 MHz, d_6_-DMSO-d_6_): 21.0, 35.9, 50.6, 52.0, 115.3, 115.8, 118.9, 126.9, 127.1, 127.8, 128.3, 129.6, 130.9, 135.3, 137.3, 143.1, 143.9, 163.5, 166.7, 170.9; HRMS (HESI^+^): [M + H]^+^: calculated for C_24_H_25_N_2_O_6_S^+^ (*m*/*z*): 469.1433, found 469.1430.

*4-(N-(Benzyl)-2-(2,3,4,5,6-pentafluoro-N-methylphenylsulfonamido)acetamido)-2-hydroxybenzoic acid* (**4**). Isolated in 74% yield as a pale-pink crystalline solid. Mp: 176.0 ± 1 °C, *δ*_H_ (500 MHz, DMSO-d_6_): 3.02 (3H, s, CH_3_N), 4.09 (2H, s, CH_2_), 4.75 (2H, s, CH_2_), 6.40–6.42 (1H, m, Ar), 6.47 (1H, d, *J* = 1.8 Hz, Ar), 7.12 (2H, d, *J* = 7.0 Hz, Ar), 7.22–7.30 (3H, m, Ar), 7.69 (1H, d, *J* = 8.1 Hz, Ar); *δ*_C_ (126 MHz, d_6_-DMSO-d_6_): 35.6, 51.2, 52.1, 115.4, 115.6, 119.3–119.5 (m), 127.2, 127.9, 128.2, 131.1, 136.2–138.6 (m), 136.9, 143.0–145.3 (m), 163.6, 166.1, 170.8; HRMS (HESI^+^): [M + H]^+^: calculated for C_23_H_18_F_5_N_2_O_6_S^+^ (*m*/*z*): 545.0806, found 545.0807.

*4-(N-(4-tert-Butylbenzyl)-2-(N-methyl-N-(p-toluenesulfonyl))acetamido)-2-hydroxybenzoic acid* (**5**). Isolated in 77% yield as a pale-brown solid. Mp: 136.0 ± 1 °C; *δ*_H_ (500 MHz, CD_3_OD-d_4_): 1.29 (9H, s, *t*-Bu), 2.39 (3H, s, CH_3_(tol)), 2.83 (3H, s, CH_3_N), 3.76 (2H, s, CH_2_), 4.82 (2H, s, CH_2_), 6.56–6.60 (1H, m, Ar), 6.64 (1H, s, Ar), 7.11 (2H, d, *J* = 8.3 Hz, Ar), 7.29–7.34 (4H, m, Ar), 7.56 (2H, d, *J* = 8.2 Hz, Ar), 7.87 (1H, d, *J* = 8.2 Hz, Ar); *δ*_C_ (126 MHz, CD_3_OD-d_4_): 21.5, 31.8, 35.4, 36.9, 52.6, 53.8, 117.4, 119.2, 126.4, 128.5, 129.4, 130.7, 133.1, 135.1, 136.3, 145.1, 146.8, 151.7, 163.7, 169.3; HRMS (HESI^+^): [M + H]^+^: calculated for C_28_H_33_N_2_O_6_S^+^ (*m*/*z*): 525.2059, found 525.2060.

*4-(N-(4-tert-Butylbenzyl)-2-(2,3,4,5,6-pentafluoro-N-methylphenylsulfonamido)acetamido)-2-hydroxybenzoic acid* (**6**). Isolated in 99% yield as a white solid. Mp: 87.1 ± 1 °C; *δ*_H_ (500 MHz, CDCl_3_-d): 1.30 (9H, s, *t*-Bu), 3.11 (3H, s, CH_3_N), 4.08 (2H, s, CH_2_), 4.76 (2H, s, CH_2_), 6.58–6.62 (1H, m, Ar) 6.73 (1H, d, *J* = 2.0 Hz, Ar), 7.02 (2H, d, *J* = 8.2 Hz, Ar), 7.30 (2H, d, *J* = 8.2 Hz, Ar), 7.91 (1H, d, *J* = 8.4 Hz, Ar), 10.60 (1H, s, COOH); *δ*_C_ (126 MHz, CDCl_3_-d): 31.4, 34.7, 36.1, 52.4, 53.2, 109.9, 111.9, 117.2–117.5 (m), 119.4, 125.8, 128.2, 132.8, 136.6–139.1 (m), 143.8–146.1 (m), 147.7, 151.2, 163.3, 166.2, 172.7; HRMS (HESI^+^): [M + H]^+^: calculated for C_27_H_26_F_5_N_2_O_6_S^+^ (*m*/*z*): 601.1432, found 601.1431.

*4-(N-(4-Methoxybenzyl)-2-(2,4,6-trifluoro-N-methylphenylsulfonamido)acetamido)-2-hydroxybenzoic acid* (**7**). Isolated in 81% as a white crystalline solid. Mp: 85.5 ± 1 °C; *δ*_H_ (500 MHz, CD_3_OD-d_4_): 3.05 (3H, s, CH_3_N), 3.76 (3H, s, CH_3_O), 4.03 (2H, s, CH_2_), 4.75 (2H, s, CH_2_), 6.61 (1H, dd, *J* = 8.4 Hz, *J* = 2.0 Hz, Ar), 6.67 (1H, d, *J* = 2.0 Hz, Ar), 6.80–6.84 (2H, m, Ar), 6.99–7.06 (4H, m, Ar), 7.86 (1H, d, *J* = 8.0 Hz, Ar); *δ*_C_ (126 MHz, CD_3_OD-d_4_): 36.3, 52.6, 53.3, 55.7, 103.0 (td, *J* = 29.0 Hz, *J* = 4.0 Hz), 114.2, 114.9, 118.1, 120.1, 129.8, 131.0, 133.0, 147.8, 160.7, 162.0 (ddd, *J* = 257.7 Hz, *J* = 15.7 Hz, *J* = 7.0 Hz), 164.0, 165.3–167.6 (m), 168.3, 172.7; HRMS (HESI^+^): [M + H]^+^: calculated for C_24_H_22_F_3_N_2_O_7_S^+^ (*m*/*z*): 539.1055, found 539.1083.

*4-(N-(4-Methoxybenzyl)-2-(2,3,4,5,6-pentafluoro-N-methylphenylsulfonamido)acetamido)-2-hydroxybenzoic acid* (**8**). Isolated in 72% yield as a white crystalline solid. Mp: 89.0 ± 1 °C; *δ*_H_ (500 MHz, CD_3_OD-d_4_): 3.07 (3H, s, CH_3_N), 3.76 (3H, s, CH_3_O), 4.08 (2H, s, CH_2_), 4.74 (2H, s, CH_2_), 6.60 (1H, dd, *J* = 8.3 Hz, *J* = 1.1 Hz, Ar), 6.67 (1H, s, Ar), 6.81 (2H, d, *J* = 8.2 Hz, Ar), 7.03 (2H, d, *J* = 8.2 Hz, Ar), 7.87 (1H, d, *J* = 8.3 Hz, Ar); *δ*_C_ (126 MHz, CD_3_OD-d_4_): 36.4, 52.9, 53.3, 55.6, 114.3, 114.9, 118.1, 120.1, 129.7, 131.0, 133.1, 138.1–140.4 (m), 143.9–145.3 (m), 147.6, 160.8, 164.0, 168.0, 172.7; HRMS (HESI^+^): [M + H]^+^: calculated for C_24_H_20_F_5_N_2_O_7_S^+^ (*m*/*z*): 575.0867, found: 575.0914.

*4-(N-(4-Trifluoromethylbenzyl)-2-(N-methylphenylsulfonyl)acetamido)-2-hydroxybenzoic acid* (**9**). Isolated in 59% yield as a pale-brown solid. Mp: 81.5 ± 1 °C; *δ*_H_ (500 MHz, CD_3_OD-d_4_): 2.87 (3H, s, CH_3_N), 3.85 (2H, s, CH_2_), 4.96 (2H, s, CH_2_), 6.72 (1H, dd, *J* = 8.4 Hz, *J* = 2.1 Hz, Ar), 6.80 (1H, d, *J* = 2.0 Hz, Ar), 7.41 (2H, d, *J* = 8.1 Hz, Ar), 7.49–7.53 (2H, m, Ar), 7.58–7.62 (3H, m, Ar), 7.70–7.72 (2H, m, Ar), 7.89 (1H, d, *J* = 8.4 Hz, Ar); *δ*_C_ (126 MHz, CD_3_OD-d_4_): 36.9, 52.6, 53.6, 114.3, 117.8, 119.8, 125.6 (q, *J* = 271.6 Hz), 126.5 (q, *J* = 3.8 Hz), 128.4, 130.0, 130.2, 130.8 (q, *J* = 32.4 Hz), 133.2, 134.0, 139.4, 142.6, 148.0, 164.0, 169.4, 172.6; HRMS (HESI^+^): [M + H]^+^: calculated for C_24_H_22_F_3_N_2_O_6_S^+^ (*m*/*z*): 523.1151, found 523.1153.

*4-(N-(4-Trifluoromethylbenzyl)-2-(N-methyl-N-(p-toluenesulfonyl))acetamido)-2-hydroxybenzoic acid* (**10**). Isolated in 72% yield as a white solid. Mp: 176.5 ± 1 °C; *δ*_H_ (500 MHz, CD_3_OD-d_4_): 2.39 (3H, s, CH_3_(tol.)), 2.84 (3H, s, CH_3_N), 3.82 (2H, s, CH_2_), 4.96 (2H, s, CH_2_), 6.71 (1H, dd, *J* = 8.4 Hz, *J* = 2.1 Hz, Ar), 6.80 (1H, d, *J* = 2.1 Hz, Ar), 7.31 (2H, d, *J* = 8.1 Hz, Ar), 7.41 (2H, d, *J* = 8.1 Hz, Ar), 7.56–7.61 (4H, m, Ar), 7.88 (1H, d, *J* = 8.4 Hz, Ar); *δ*_C_ (126 MHz, CD_3_OD-d_4_): 21.4, 36.9, 52.6, 53.6, 114.2, 117.8, 119.8, 125.6 (q, *J* = 271.3 Hz), 126.4 (q, *J* = 3.6 Hz), 128.5, 130.0, 130.7, 130.8 (q, *J* = 32.3 Hz), 133.2, 136.3, 142.6, 145.1, 148.1, 164.0, 169.5, 172.6; HRMS (HESI^+^): [M + H]^+^: calculated for C_25_H_24_F_3_N_2_O_6_S^+^ (*m*/*z*): 537.1307, found 537.1307.

*4-(N-(4-Trifluoromethylbenzyl)-2-(4-fluoro-N-methylphenylsulfonamido)acetamido)-2-hydroxybenzoic acid* (**11**). Isolated in 57% as a white solid. Mp: 218.0 ± 1 °C; *δ*_H_ (500 MHz, CD_3_OD-d_4_): 2.88 (3H, s, CH_3_N), 3.89 (2H, s, CH_2_), 4.95 (2H, s, CH_2_), 6.72 (1H, dd, *J* = 8.4 Hz, *J* = 2.0 Hz, Ar), 6.80 (1H, d, *J* = 2.0 Hz, Ar), 7.25 (2H, tt, *J* = 8.8 Hz, *J* = 2.0 Hz, Ar), 7.40 (2H, d, *J* = 8.1 Hz, Ar), 7.60 (2H, d, *J* = 8.2 Hz, Ar), 7.77–7.81 (2H, m, Ar), 7.90 (1H, d, *J* = 8.4 Hz, Ar); *δ*_C_ (126 MHz, CD_3_OD-d_4_): 36.7, 52.5, 53.6, 114.4, 117.2 (d, *J* = 22.9 Hz), 117.8, 119.8, 125.6 (q, *J* = 271.0 Hz), 126.5 (q, *J* = 3.7 Hz), 130.1, 130.8 (q, *J* = 32.0 Hz), 131.4 (d, *J* = 9.5 Hz), 133.2, 135.9 (d, *J* = 3.1 Hz), 142.6, 147.9, 164.1, 166.6 (d, *J* = 253.0 Hz), 169.3, 172.7; HRMS (HESI^+^): [M + H]^+^: calculated for C_24_H_21_F_4_N_2_O_6_S^+^ (*m*/*z*): 541.1056, found 541.1057.

*4-(N-(4-Trifluoromethylbenzyl)-2-(3,5-difluoro-N-methylphenylsulfonamido)acetamido)-2-hydroxybenzoic acid* (**12**). Isolated in quantitative yield as a white solid. Mp: 82.1 ± 1 °C; *δ*_H_ (500 MHz, CD_3_OD-d_4_): 2.94 (3H, s, CH_3_N), 3.95 (2H, s, CH_2_), 4.94 (2H, s, CH_2_), 6.72 (1H, dd, *J* = 8.4 Hz, *J* = 2.0 Hz, Ar), 6.81 (1H, d, *J* = 2.0 Hz, Ar), 7.26 (1H, tt, *J* = 9.0 Hz, *J* = 2.2 Hz, Ar), 7.36–7.41 (4H, m, Ar), 7.60 (2H, d, *J* = 8.1 Hz, Ar), 7.90 (1H, d, *J* = 8.4 Hz, Ar); *δ*_C_ (126 MHz, CD_3_OD-d_4_): 36.8, 52.5, 53.5, 109.2 (t, *J* = 25.7 Hz), 111.8–112.2 (m), 114.4, 117.8, 119.8, 125.6 (q, *J* = 271.0 Hz), 126.5 (q, *J* = 3.8 Hz), 130.0, 130.9 (q, *J* = 32.3 Hz), 133.3, 142.5, 143.4 (t, *J* = 8.4 Hz), 147.8, 164.1, 164.3 (dd, *J* = 252.4 Hz, *J* = 12.0 Hz), 169.0, 172.6; HRMS (HESI^+^): [M + H]^+^: calculated for C_24_H_20_F_5_N_2_O_6_S^+^ (*m*/*z*): 559.0962, found 559.0963.

*4-(N-(4-Trifluoromethylbenzyl)-2-(2,4,6-trifluoro-N-methylphenylsulfonamido)acetamido)-2-hydroxybenzoic acid* (**13**). Isolated in 99% yield as a white solid. Mp: 85.5 ± 1 °C; *δ*_H_ (500 MHz, CD_3_OD-d_4_): 3.05 (3H, s, CH_3_N), 4.09 (2H, s, CH_2_), 4.91 (2H, s, CH_2_), 6.69 (1H, dd, *J* = 8.3 Hz, *J* = 2.0 Hz, Ar), 6.78 (1H, d, *J* = 2.0 Hz, Ar), 7.00 (2H, t, *J* = 9.3 Hz, Ar), 7.37 (2H, d, *J* = 8.1 Hz, Ar), 7.60 (2H, d, *J* = 8.2 Hz, Ar), 7.88 (1H, d, *J* = 8.4 Hz, Ar); *δ*_C_ (126 MHz, CD_3_OD-d_4_): 36.3, 52.6, 53.5, 102.7–103.2 (m), 114.5, 115.6 (td, *J* = 17.2 Hz, *J* = 5.3 Hz), 117.8, 119.8, 125.6 (q, *J* = 271.1 Hz), 126.4 (q, *J* = 3.5 Hz), 130.1, 130.8 (q, *J* = 32.2 Hz), 133.3, 142.5, 147.7, 162.0 (ddd, *J* = 257.5 Hz, *J* = 15.6 Hz, *J* = 6.9 Hz), 164.1, 166.5 (dt, *J* = 255.3 Hz, *J* = 16.1 Hz), 168.8, 172.6; HRMS (HESI^+^): [M + H]^+^: calculated for C_24_H_19_F_6_N_2_O_6_S^+^ (*m*/*z*): 577.0868, found 577.0869.

*4-(N-(4-Trifluoromethylbenzyl)-2-(2,3,4,5,6-pentafluoro-N-methylphenylsulfonamido)acetamido)-2-hydroxybenzoic acid* (**14**). Isolated in 93% yield as a white solid. Mp: 154.5 ± 1 °C; *δ*_H_ (500 MHz, CD_3_OD-d_4_): 3.08 (3H, s, CH_3_N), 4.15 (2H, s, CH_2_), 4.91 (2H, s, CH_2_), 6.69 (1H, dd, *J* = 8.3 Hz, *J* = 2.0 Hz, Ar), 6.78 (1H, d, *J* = 2.0 Hz, Ar), 7.36 (2H, d, *J* = 8.1 Hz, Ar), 7.59 (2H, d, *J* = 8.2 Hz, Ar), 7.91 (1H, d, *J* = 8.4 Hz, Ar); *δ*_C_ (126 MHz, CD_3_OD-d_4_): 36.4, 52.9, 53.6, 114.6, 117.8, 119.8, 126.4–126.5 (m), 130.0, 130.9 (q, *J* = 32.0 Hz), 133.4, 138.0–140.6 (m), 142.4, 143.8–146.3 (m), 144.9–147.3 (m), 147.5, 164.2, 168.5, 172.6; HRMS (HESI^+^): [M + H]^+^: calculated for C_24_H_17_F_8_N_2_O_6_S^+^ (*m*/*z*): 613.0680, found 613.0680.

### 4.3. Antibodies

The following primary antibodies were used: mouse monoclonal STAT3 (Santa Cruz Biotechnology, Inc., Dallas, TX, cat. no. sc-8019; dilution 1:1000), mouse monoclonal pSTAT3 (Y705; Cell Signaling Technology, Danvers, MA, cat. no. 9138; dilution 1:500), mouse monoclonal GAPDH (Santa Cruz, cat. no. sc-47724; dilution 1:10,000), rabbit monoclonal anti-mouse STAT3 (Cell Signaling Technology, Danvers, MA, cat. no. 4904; dilution 1:2000), rabbit monoclonal anti-mouse pSTAT3 (Y705; Cell Signaling Technology, Danvers, MA, cat. no. 9145S; dilution 1:2000), and rabbit monoclonal anti-mouse GAPDH (Cell Signaling Technology, Danvers, MA, cat. no. 2118S; dilution 1:10,000). The following secondary antibodies were used: goat anti-mouse HRP-conjugated (Bio-Rad, Hercules, CA, cat. no. 170–6516; dilution 1:10,000) and goat IgG-HRP anti-rabbit (Cell Signaling Technology, Danvers, MA, cat. no. 7074S; dilution 1:2000).

### 4.4. Cell cultures

All cell lines used in the study were obtained from the American Type Culture Collection (ATCC, Manassas, VA). Human breast adenocarcinoma MDA-MB-231, prostate carcinoma DU-145, cervix carcinoma HeLa, osteosarcoma U2-OS, glioblastoma U373, U-87 MG and T98-G, and telomerase-immortalised retinal epithelium RPE-1-hTERT cell lines were cultured in high-glucose (4.5 g/l) DMEM. Media were supplemented with 10% foetal bovine serum (Gibco/Thermo Fisher Scientific, Waltham, MA), 100 U/ml penicillin, and 100 μg/ml streptomycin sulphate (Gibco/Thermo Fisher Scientific, Waltham, MA). Cells were kept at 37 °C under 5% CO_2_ atmosphere and 95% humidity.

The mouse TC-1 cell line was obtained *in vitro* by co-transfection of murine lung C57BL/6 cells with HPV16 *E6/E7* and activated human *H*-*Ras* (G12V) oncogenes. TC-1 cells were cultured in RPMI-1640 medium supplemented with 10% foetal calf serum, l-glutamine, and antibiotics^35^. Prostate carcinoma cell line TRAMP-C2^36^ was maintained in DMEM medium (Sigma-Aldrich, St. Louis, MO) supplemented with 5% FCS, Nu-Serum IV (5%; BD Biosciences, Bedford, MA), 5 µg/ml human insulin (Sigma-Aldrich, St. Louis, MO), dehydroisoandrosterone (DHEA, 10 nM; Sigma-Aldrich, St. Louis, MO), and antibiotics^37^.

### 4.5. Resazurin assay and IC_50_ cytotoxicity estimation

The resazurin reduction assay^34^ was performed as described previously^38^. Briefly, cells were seeded onto a 96-well plate (2000 cells per well) in hexaplicate and the following day treated with the tested compounds (dissolved in DMSO as 50 mM stock) in a concentration range as indicated. After 24 h, 200 μl of culture medium of each well was exchanged for 100 μl of resazurin (stock 30 mg/ml; Sigma, St. Louis, MO) diluted 10 times in the culture medium, and the cells were incubated at 37 °C for 1–3 h. Reading of fluorescence was done using an Envision reader (PerkinElmer, Waltham, MA). Absolute values of fluorescence were related to the values of control/untreated cells. To estimate the cytotoxic concentration (half maximal inhibitory concentration, IC_50_), log-scale data were processed in GraphPad Prism software (La Jolla, CA) using nonlinear fit curve.

### 4.6. Crystal violet assay

To determine cell viability by crystal violet assay^39^, cells were seeded in 96-well plates at densities of 3000 (RPE-1), 5000 (U373 and T98-G), and 8000 (U-87 MG) cells per well. The next day the cells were treated with the tested compounds in triplicate in a concentration range as indicated. After 24 h, the cells in each well were washed twice with 150 µl PBS and then stained in 30 µl 0.5% w/v crystal violet in 20% methanol for 15 min. Plates were washed three times with double distilled H_2_O and left to dry overnight. Crystal violet was solubilised with 75 µl 0.2% Triton X-100 (Sigma, St. Louis, MO) in PBS for 15 min. Absorbance of crystal violet was measured at 595 nm using a microplate reader (Multiskan EX, Thermo Electron Corporation, Waltham, MA). Absorbance of the treated samples was expressed as a percentage of absorbance of untreated cells.

### 4.7. MTT assay and IC_50_ cytotoxicity estimation

Cells were seeded at the density of 2000 cells per well (96-well F microplates; Nunc, Roskilde, Denmark). The following day, the tested compounds were added. In the next 24 h, MTT (3-(4,5-dimethylthiazol-2-yl)-2,5-diphenyltetrazolium bromide) was added. After 6 h, DMF (dimethylformamide) was added, and after additional 24 h, absorbance was measured at 560 nm by a microplate reader. Absorbance of the treated samples was expressed as a percentage of absorbance of untreated cells. To estimate the cytotoxic concentration (half maximal inhibitory concentration, IC_50_), log-scale data were processed in GraphPad Prism software (La Jolla, CA) using a nonlinear fit curve.

### 4.8. Detection of apoptosis by annexin V and propidium iodide staining

Cells were harvested by centrifugation, resuspended in cold PBS and again centrifuged. Cell pellets were then resuspended in Annexin V Binding Buffer (ApoFlowEx FITC Kit, ED7044, Exbio, Vestec, Czech Republic), annexin V-APC and PI were added to the cell pellet and mixed. Cells were incubated in the dark at room temperature for 15 min. After the incubation, cells were centrifuged, resuspended in Annexin V Binding Buffer and analysed by flow cytometry. Five thousand cells per sample were measured by analysing annexin V and PI using a BD FACSVerse™ flow cytometer (BD Biosciences, Franklin Lakes, NJ). Data were analysed using FlowJo 10 software (FlowJo LLC, Ashland, OR), and the percentage of viable (annexin V-negative/PI-negative) cells relative to control was plotted.

### 4.9. SDS-PAGE and immunoblotting

Cells were washed three times with PBS, harvested into 95 °C-heated Laemmli SDS sample lysis buffer (2% SDS, 50 mM Tris–Cl, 10% glycerol in double distilled H_2_O) and sonicated at 3 μm amplitude for 3 × 10 or 3 × 30 s with 10 or 30 s cooling intervals in a Soniprep 150 (MSE, London, UK) or Diagenode Bioruptor 300 (Diagenode, Liege, Belgium) for human and murine cell lines, respectively. Concentration of proteins was estimated by the Pierce^TM^ BCA Protein Assay (Thermo Scientific, Waltham, MA). DTT (100 mM) and 0.01% bromophenol blue were added to the lysates before separation by SDS-PAGE (10% gels were used). Equal protein amounts (40 or 35 μg for human or murine cell lines, respectively) were loaded into each well. Proteins were electrotransferred onto a nitrocellulose membrane (Amersham^TM^ Protran^TM^ 0.45 µm NC, GE Healthcare, Chalfont St Giles, UK) using wet transfer and detected by specific antibodies (at 4 °C overnight) combined with HRP-conjugated secondary antibodies. Peroxidase activity was detected by Amersham^TM^ ECL Western Blotting Detection Reagents (GE Healthcare, Chalfont St Giles, UK). GAPDH was used as a loading control.

### 4.10. Data processing and statistical analysis

Graphs in the figures were generated using GraphPad Prism 5, Version 5.04 (GraphPad Software, Inc., La Jolla, CA). Two-tailed paired Student's *t*-test was used to calculate *p* values and to determine statistical significance: *p* < 0.05 (*), *p* < 0.01 (**), and *p* < 0.001 (***).

## Supplementary Material

Supplemental Material
