# Supplementary material for: Design, synthesis, and in vitro evaluation of BP-1-102 analogs with modified hydrophobic fragments for STAT3 inhibition
Source: J Enzyme Inhib Med Chem. 2021 Jan 13;36(1):410–24. doi: 10.1080/14756366.2020.1871336 (PMC7808747; doi:10.1080/14756366.2020.1871336)
Supplement: Supplemental Material [file IENZ_A_1871336_SM0712.pdf]

Supplementary information

Design, synthesis and *in vitro* evaluation of BP-1-102 analogs with modified hydrophobic fragments for STAT3 inhibition

Patrik Oleksak[1⊥], Miroslav Psotka[1⊥], Marketa Vancurova[2⊥], Olena Sapega[3], Jana Bieblova[3], Milan Reinis[3], David Rysanek[2], Romana Mikyskova[3], Katarina Chalupova[1], David Malinak[1], Jana Svobodova[1], Rudolf Andrys[1], Helena Rehulkova[1], Vojtech Skopek[1], Pham Ngoc Lam[1], Jiri Bartek[2,4], Zdenek Hodny[2]*, and Kamil Musilek[1]*

[1]University of Hradec Kralove, Faculty of Science, Department of Chemistry, Rokitanskeho 62, 500 03 Hradec Kralove, Czech Republic

[2]Department of Genome Integrity and [3]Laboratory of Immunological and Tumour Models, Institute of Molecular Genetics of the Czech Academy of Sciences, Videnska 1083, 142 20 Prague, Czech Republic

[4]Genome Integrity Unit, Danish Cancer Society Research Center, Copenhagen, Denmark

⊥ These authors contributed equally to this work.

Content

1. NMR spectra of final compounds 2
2. Analysis of apoptosis by fluorescence-activated flow cytometry (FACS) and IC50 of selected compounds in MDA-MB-231 cells 16
3. Screening of BP-1-102 analogs and their cytotoxic effect on mouse TC-1 and TRAMP-C2 cells 18
4. Fluorescence-activated flow cytometry (FACS) analysis of apoptosis induced by selected compounds in TC-1 cells 19
5. Fluorescence-activated flow cytometry (FACS) analysis of apoptosis induced by selected compounds in TRAMP-C2 cells 20

1. NMR spectra of final compounds

1.1. ^{1}H and ^{13}C NMR spectrum of compound **1**

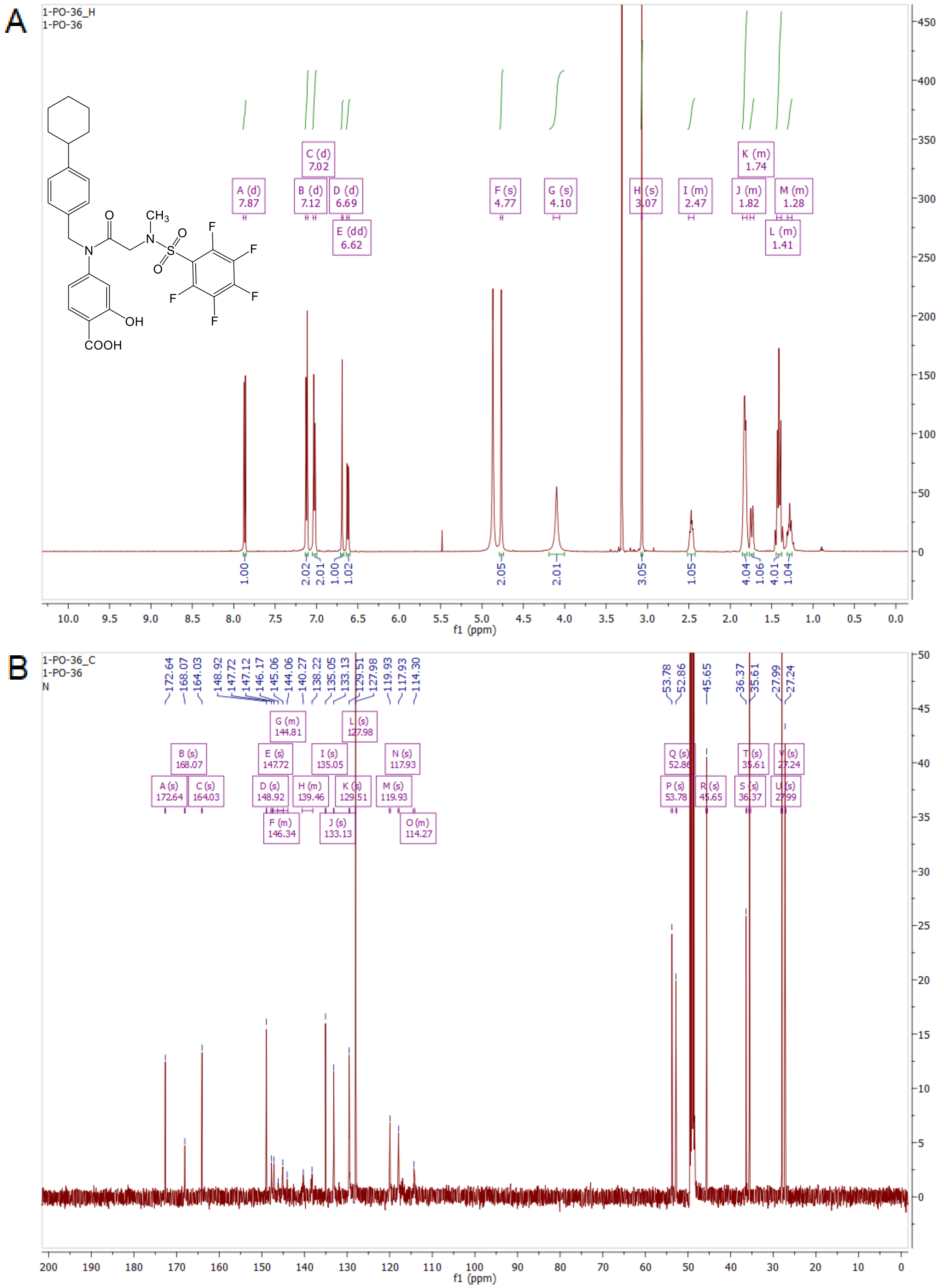

Figure S1 ^{1}H NMR spectrum, the structure (A) and ^{13}C NMR spectrum (B) of compound **1**.

1.2. ^{1}H and ^{13}C NMR spectrum of compound **2**

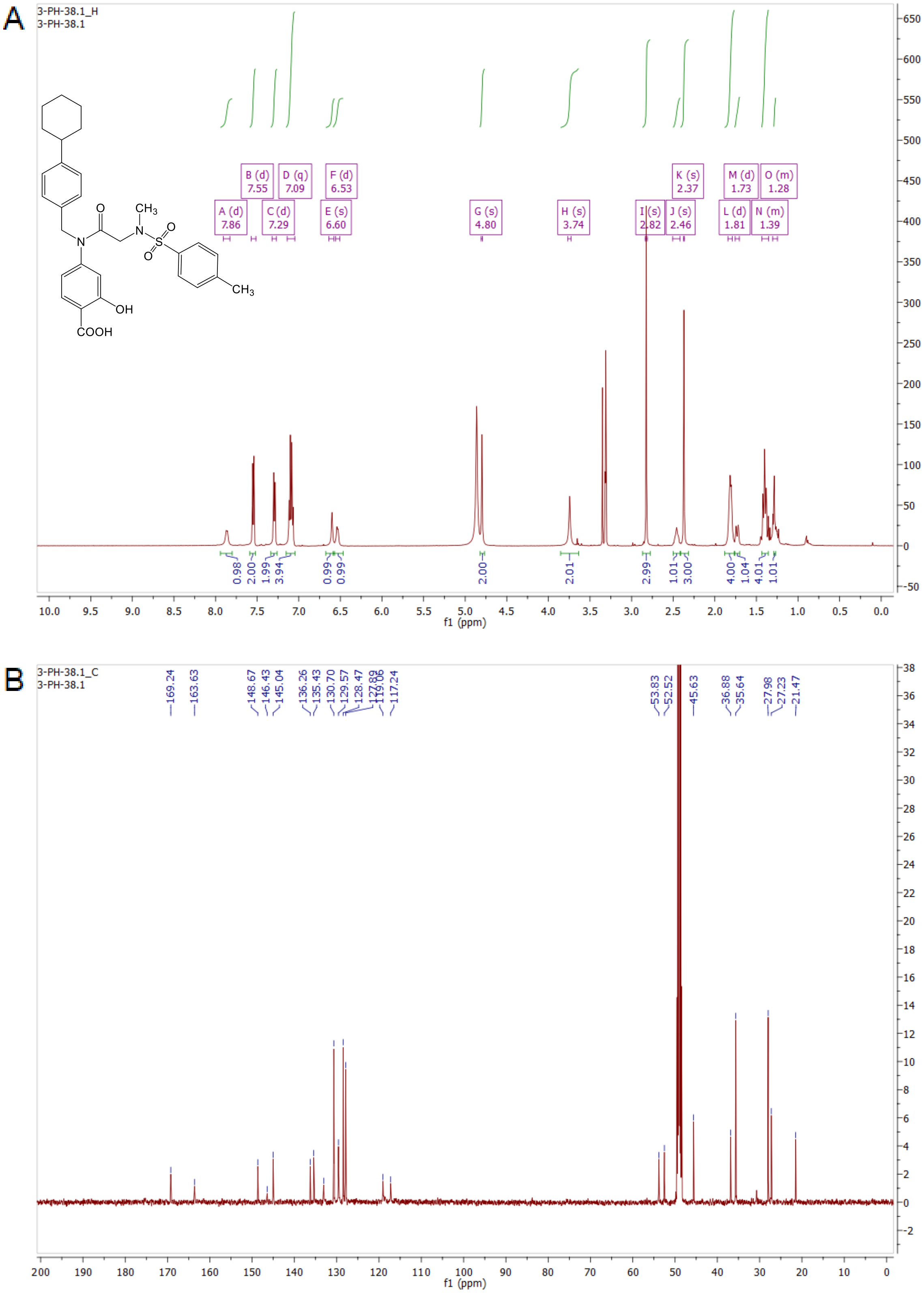

Figure S2 ^{1}H NMR spectrum, the structure (A) and ^{13}C NMR spectrum (B) of compound **2**.

1.3. ^{1}H and ^{13}C NMR spectrum of compound **3**

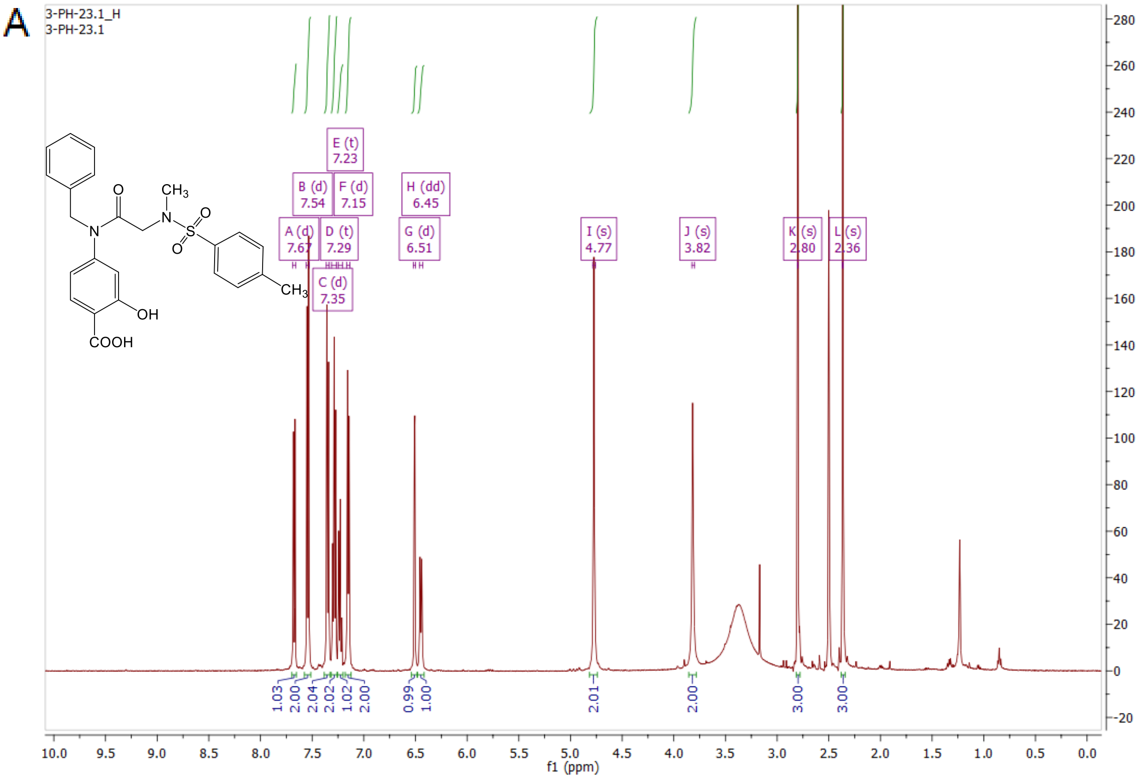

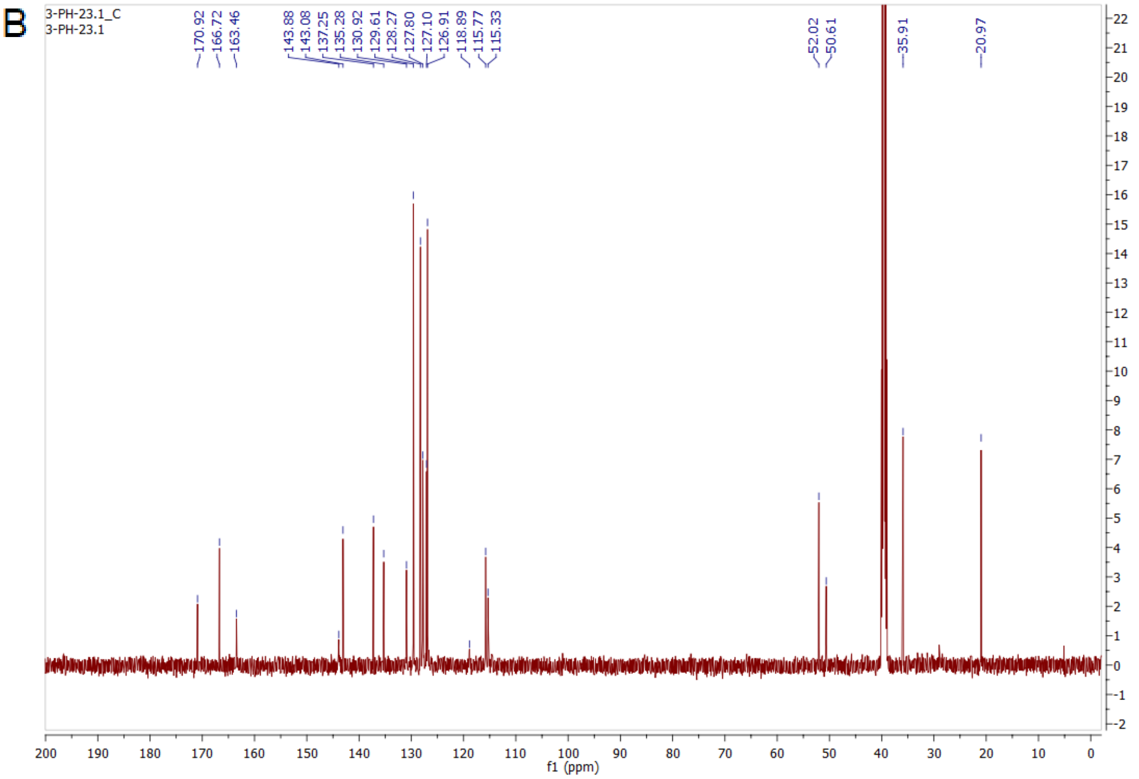

Figure S3 ^{1}H NMR spectrum, the structure (A) and ^{13}C NMR spectrum (B) of compound **3**.

1.4. ^{1}H and ^{13}C NMR spectrum of compound **4**

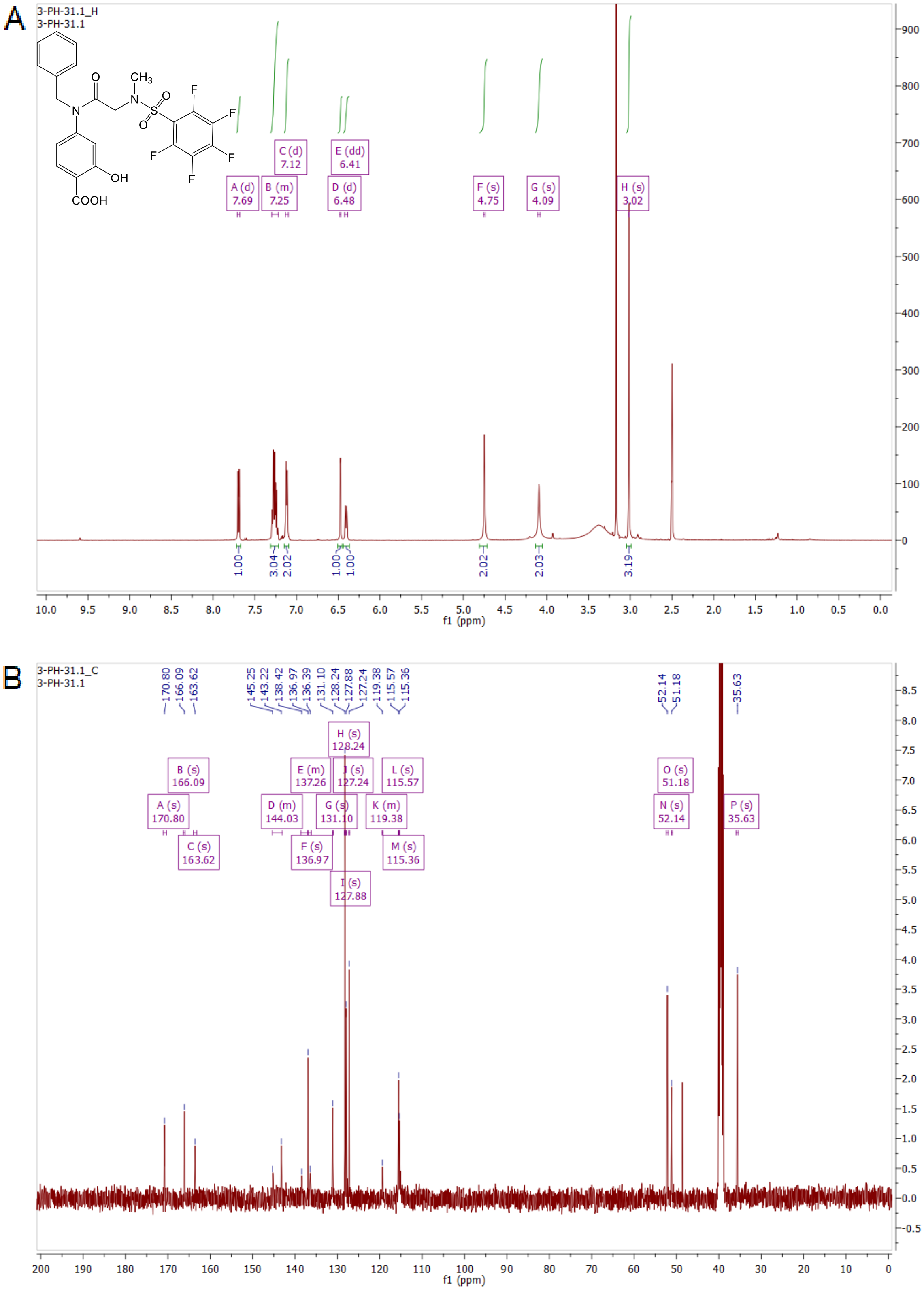

Figure S4 ^{1}H NMR spectrum, the structure (A) and ^{13}C NMR spectrum (B) of compound **4**.

1.5. ^{1}H and ^{13}C NMR spectrum of compound **5**

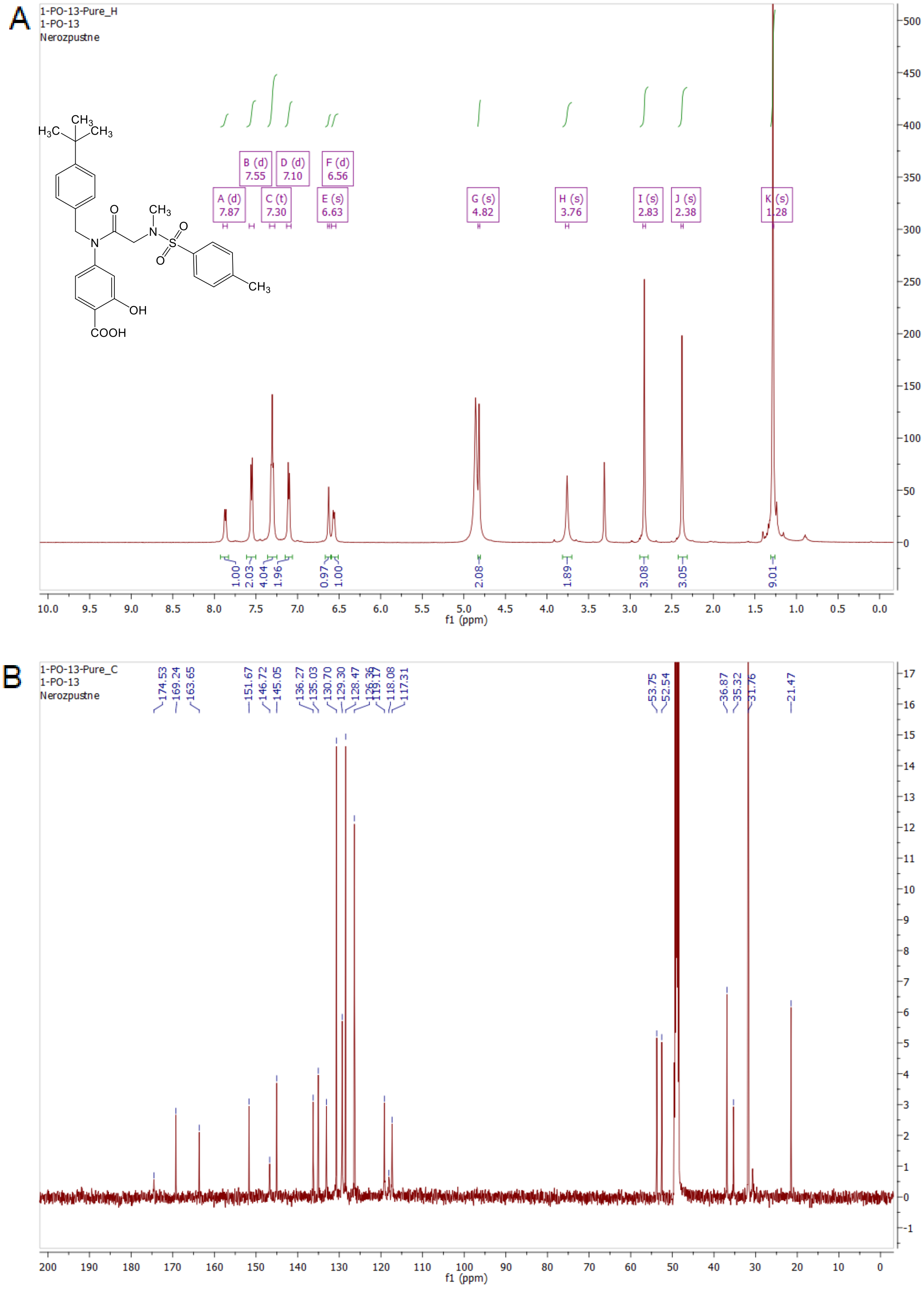

Figure S5 ^{1}H NMR spectrum, the structure (A) and ^{13}C NMR spectrum (B) of compound **5**.

1.6. ^{1}H and ^{13}C NMR spectrum of compound **6**

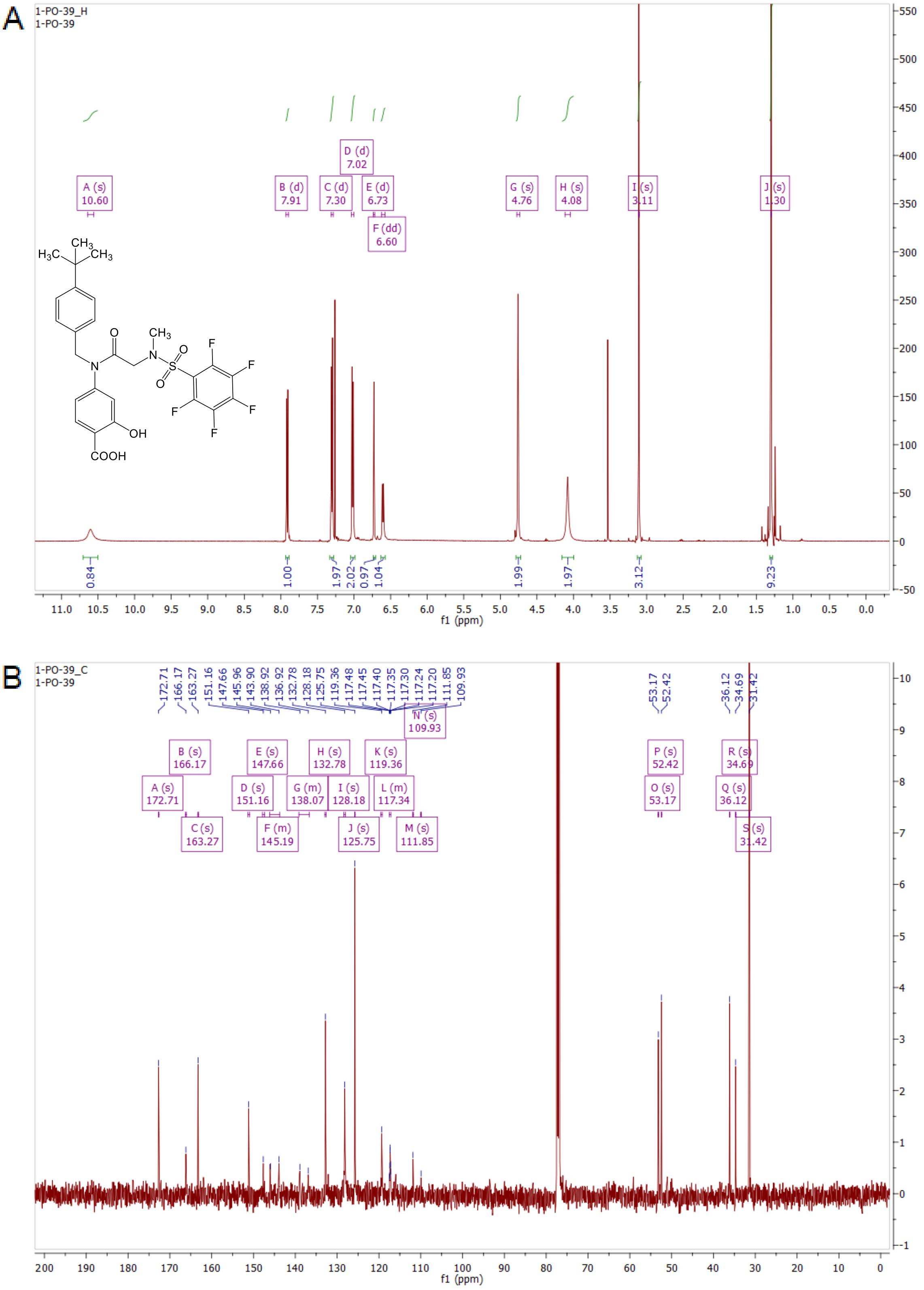

Figure S6 ^{1}H NMR spectrum, the structure (A) and ^{13}C NMR spectrum (B) of compound **6**.

1.7. ^1H and ^{13}C NMR spectrum of compound **7**

A

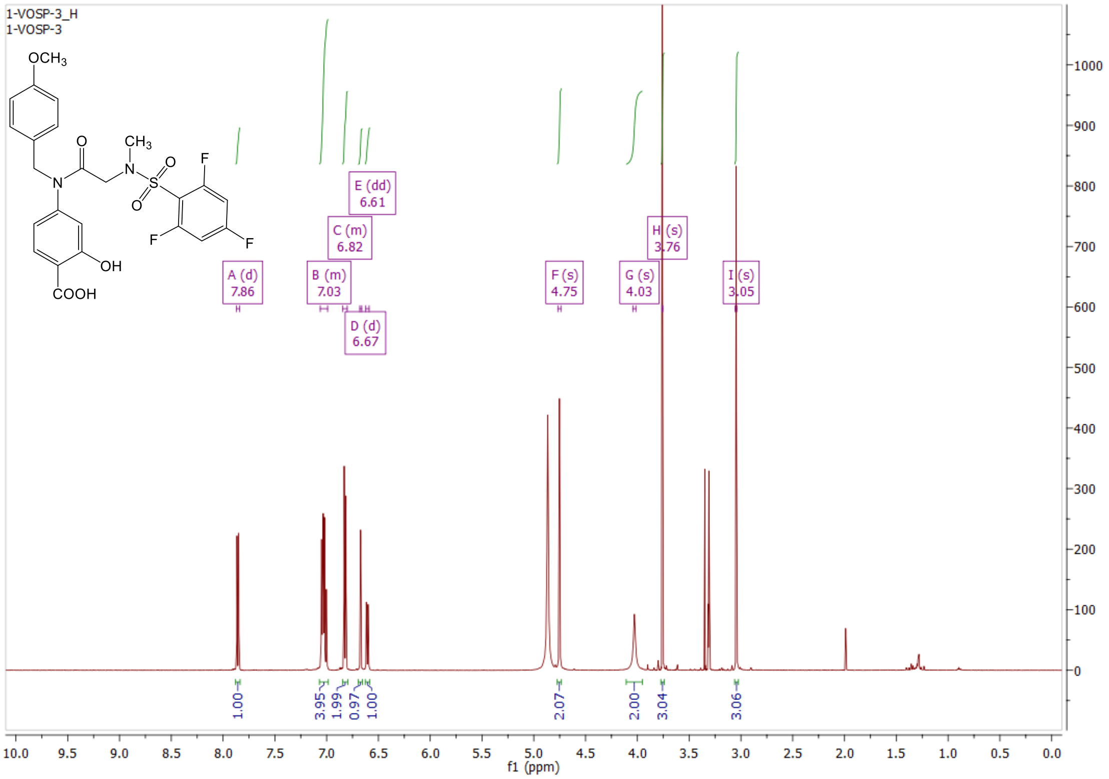

B

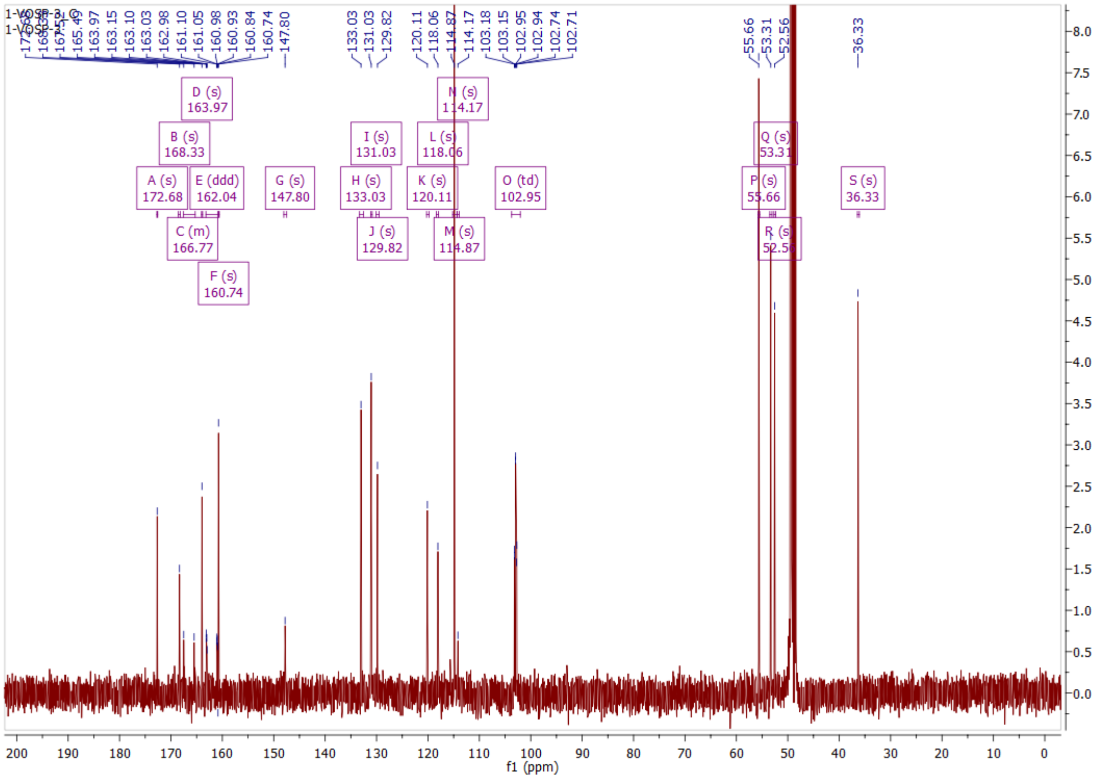

Figure S7 ^1H NMR spectrum, the structure (A) and ^{13}C NMR spectrum (B) of compound **7**.

1.8. ^{1}H and ^{13}C NMR spectrum of compound **8**

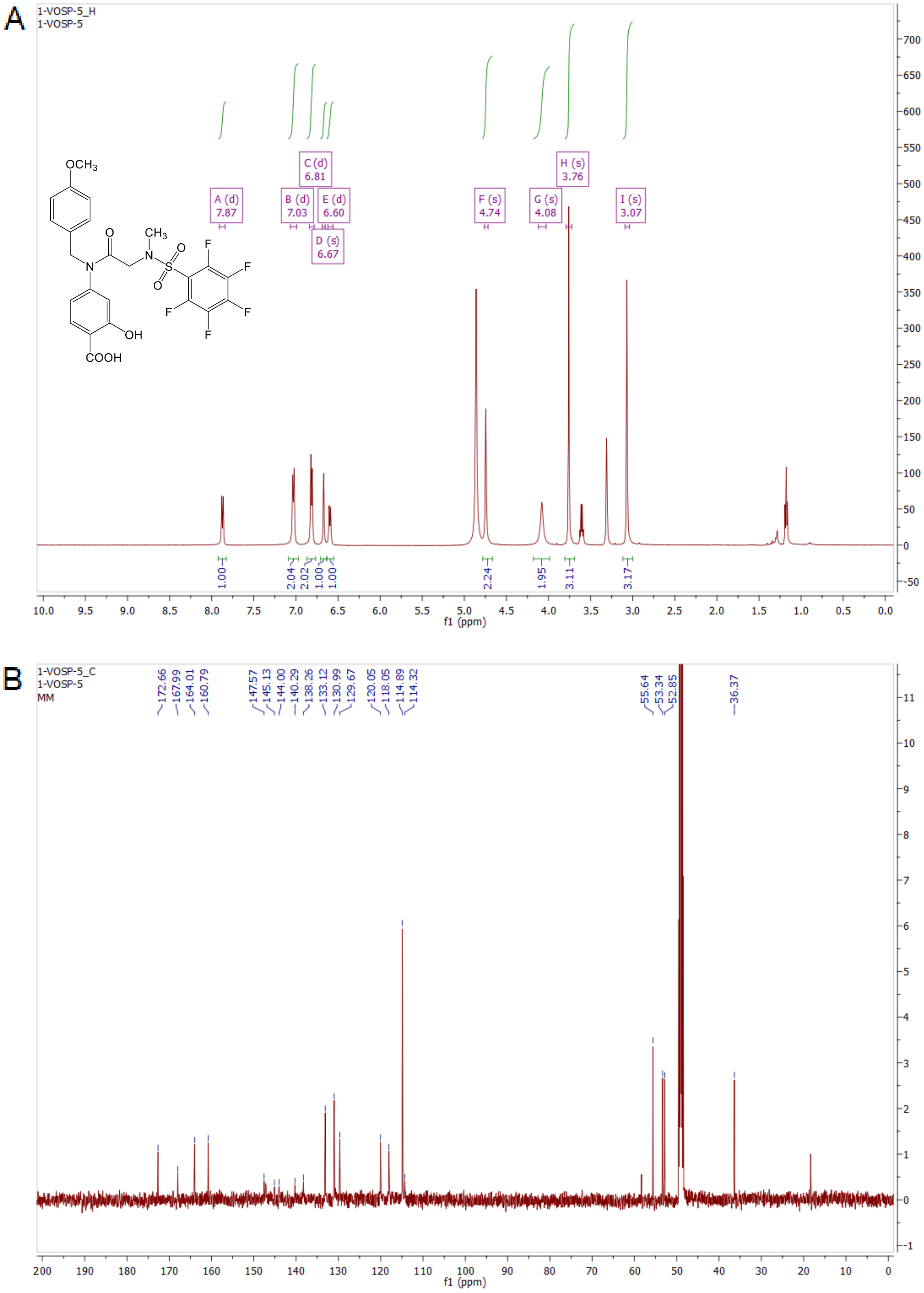

Figure S8 ^{1}H NMR spectrum, the structure (A) and ^{13}C NMR spectrum (B) of compound **8**.

1.9. ^{1}H and ^{13}C NMR spectrum of compound **9**

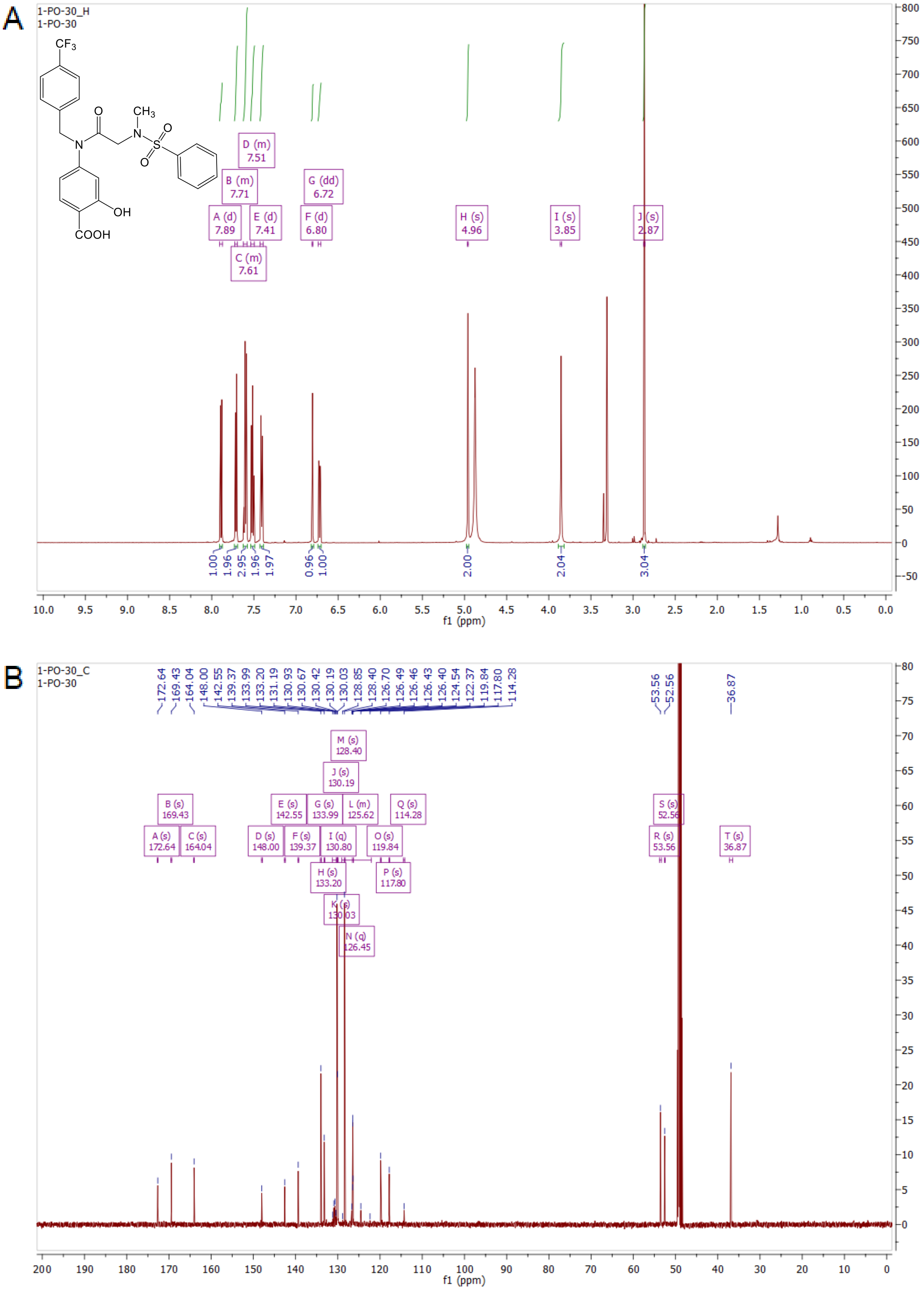

Figure S9 ^{1}H NMR spectrum, the structure (A) and ^{13}C NMR spectrum (B) of compound **9**.

1.10. ^{1}H and ^{13}C NMR spectrum of compound **10**

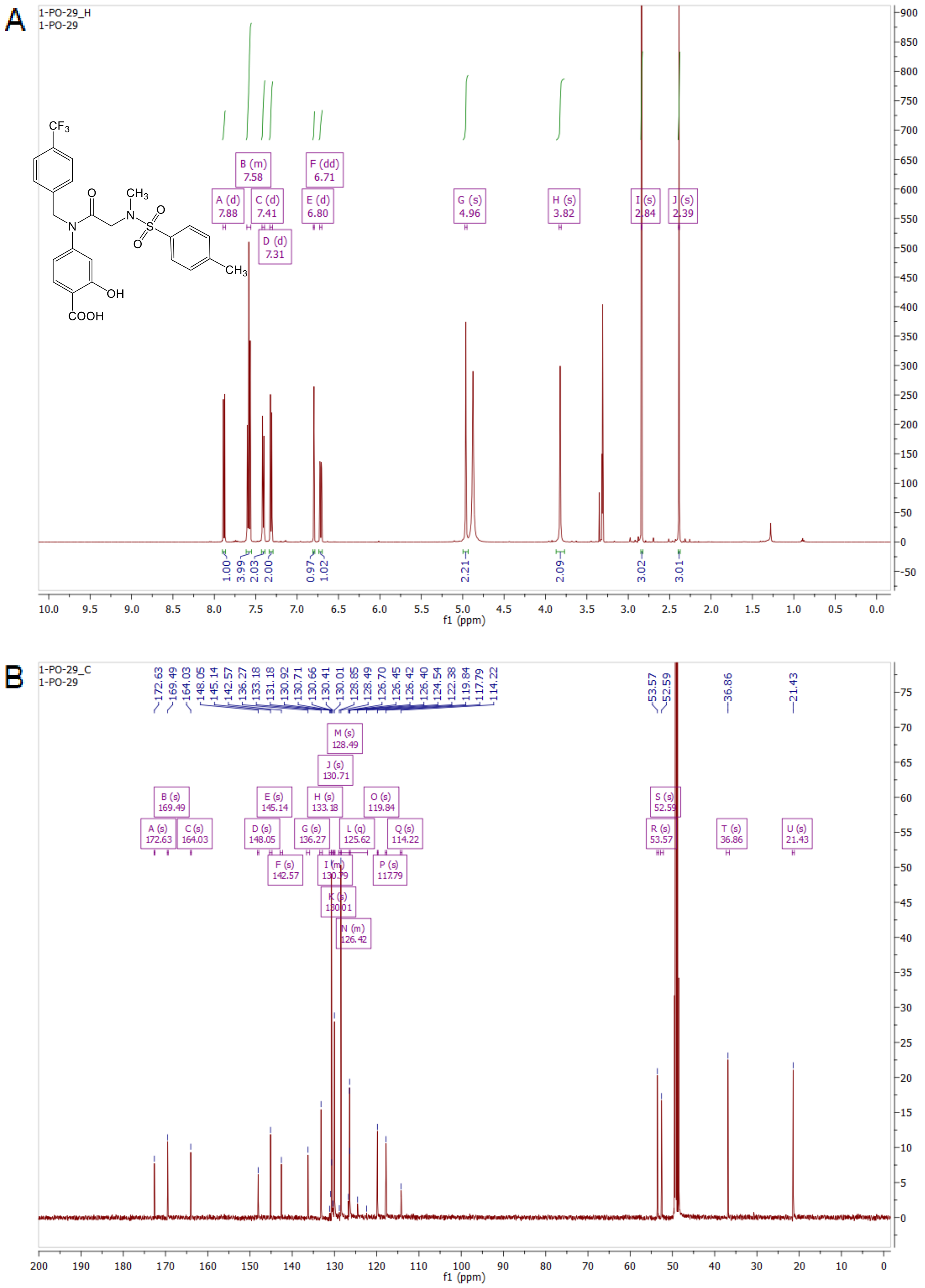

Figure S10 ^{1}H NMR spectrum, the structure (A) and ^{13}C NMR spectrum (B) of compound **10**.

1.11. ^{1}H and ^{13}C NMR spectrum of compound **11**

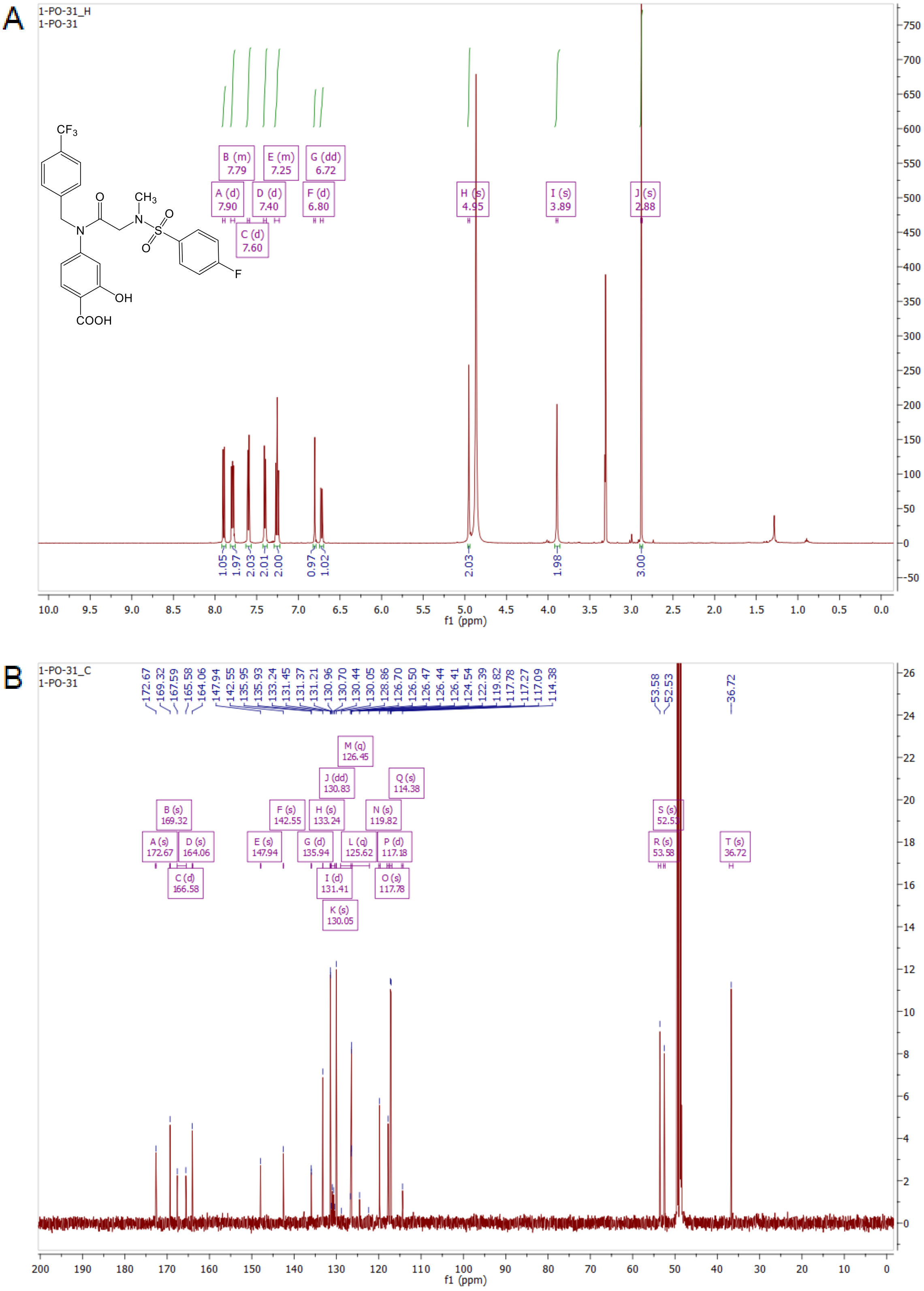

Figure S11 ^{1}H NMR spectrum, the structure (A) and ^{13}C NMR spectrum (B) of compound **11**.

1.12. ^{1}H and ^{13}C NMR spectrum of compound **12**

A

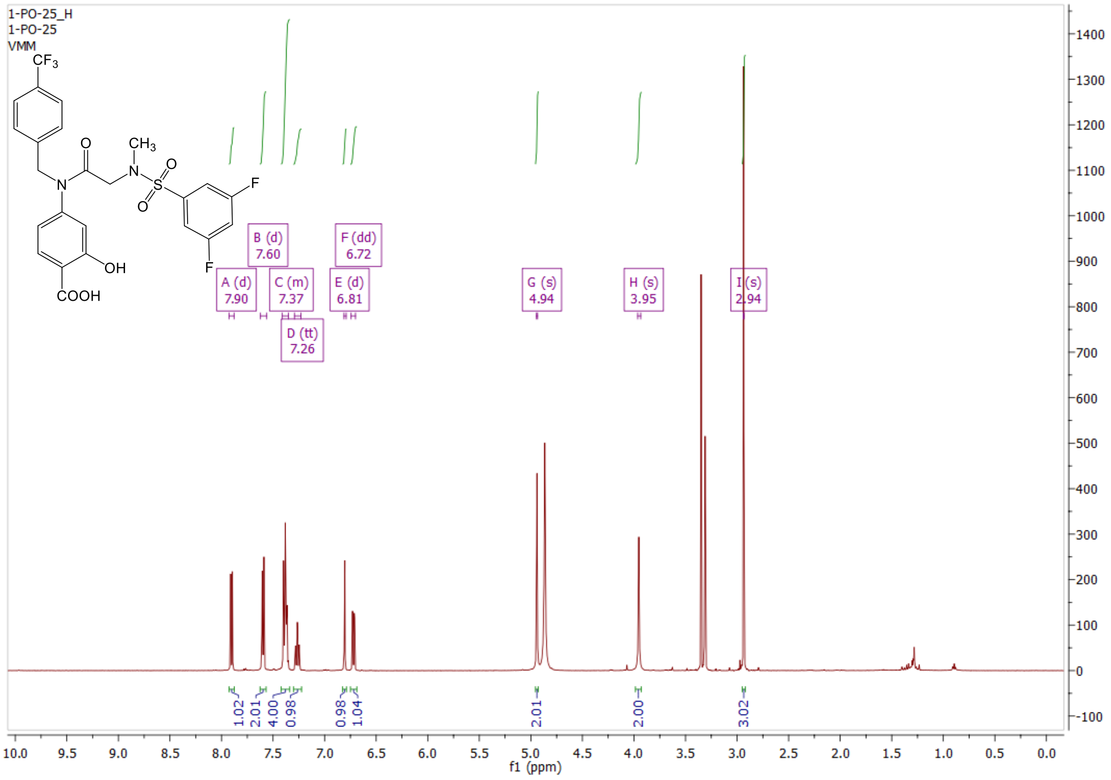

B

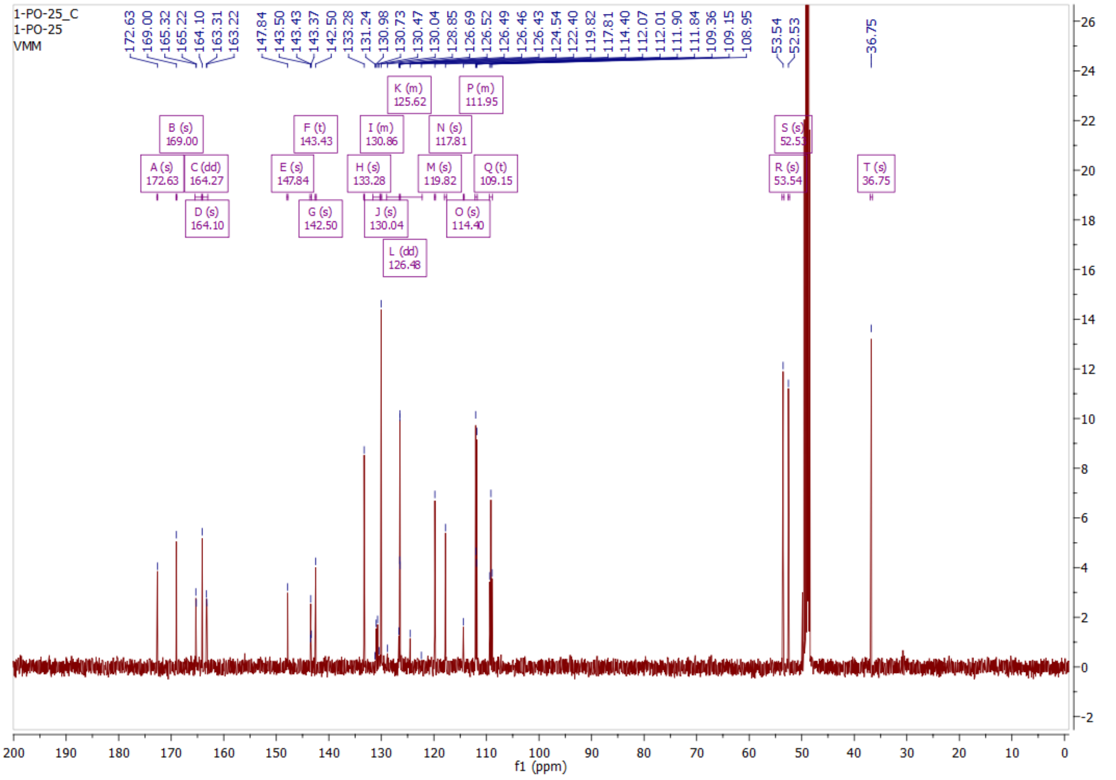

Figure S12 ^{1}H NMR spectrum, the structure (A) and ^{13}C NMR spectrum (B) of compound **12**.

1.13. ^{1}H and ^{13}C NMR spectrum of compound **13**

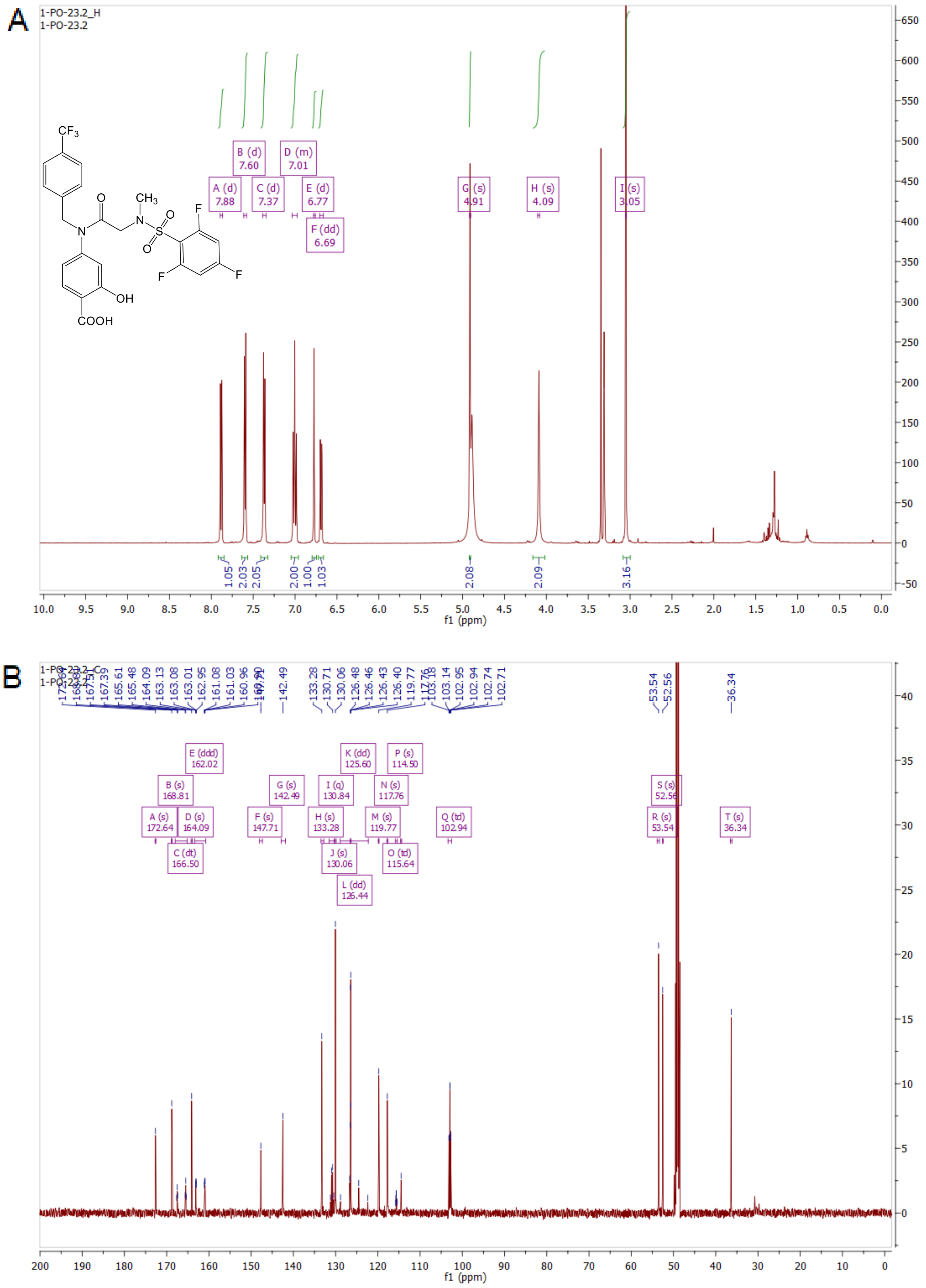

Figure S13 ^{1}H NMR spectrum, the structure (A) and ^{13}C NMR spectrum (B) of compound **13**.

1.14. ^{1}H and ^{13}C NMR spectrum of compound **14**

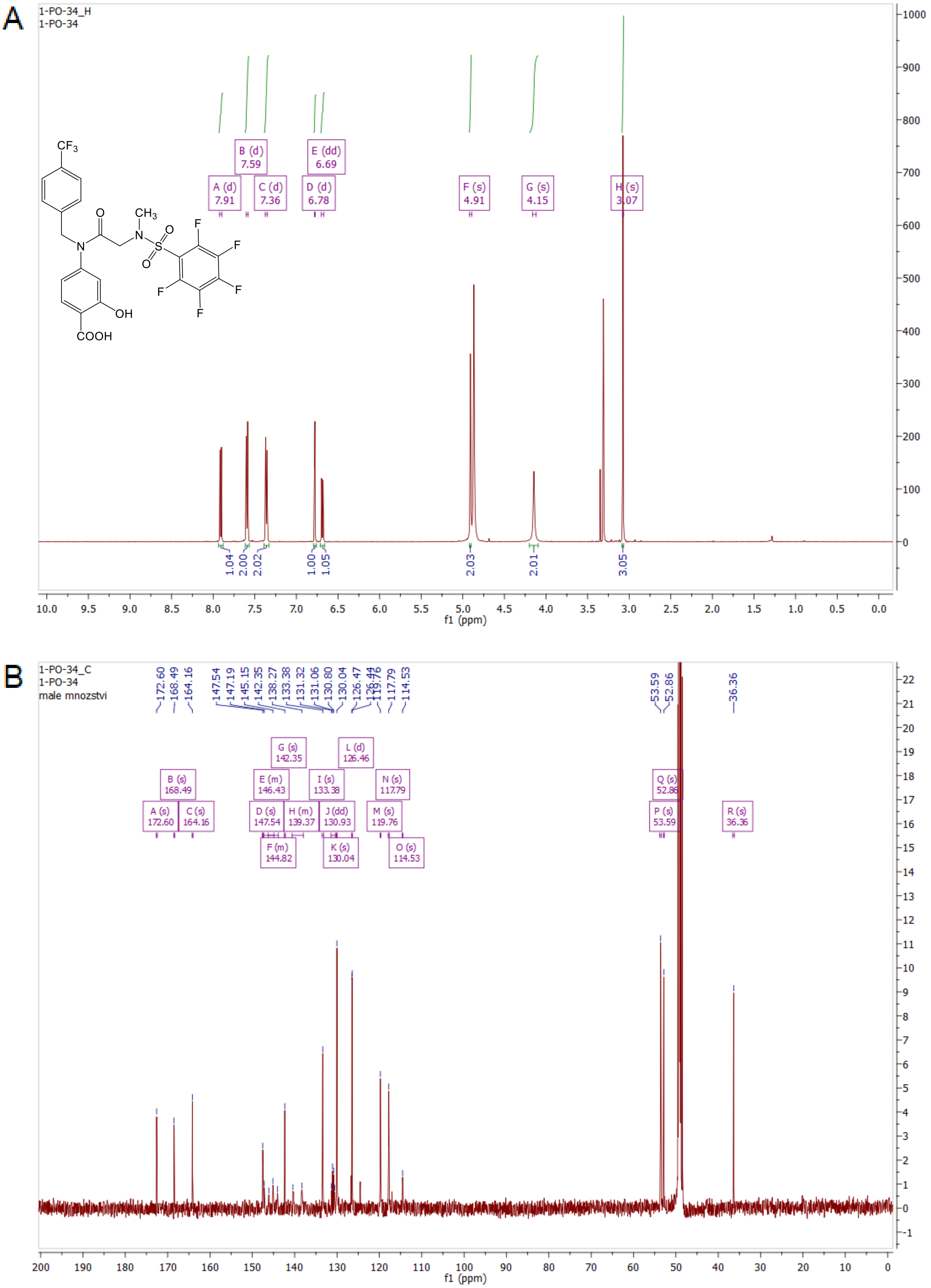

Figure S14 ^{1}H NMR spectrum, the structure (A) and ^{13}C NMR spectrum (B) of compound **14**.

2. Analysis of apoptosis by fluorescence-activated flow cytometry (FACS) and IC50 of selected compounds in MDA-MB-231 cells

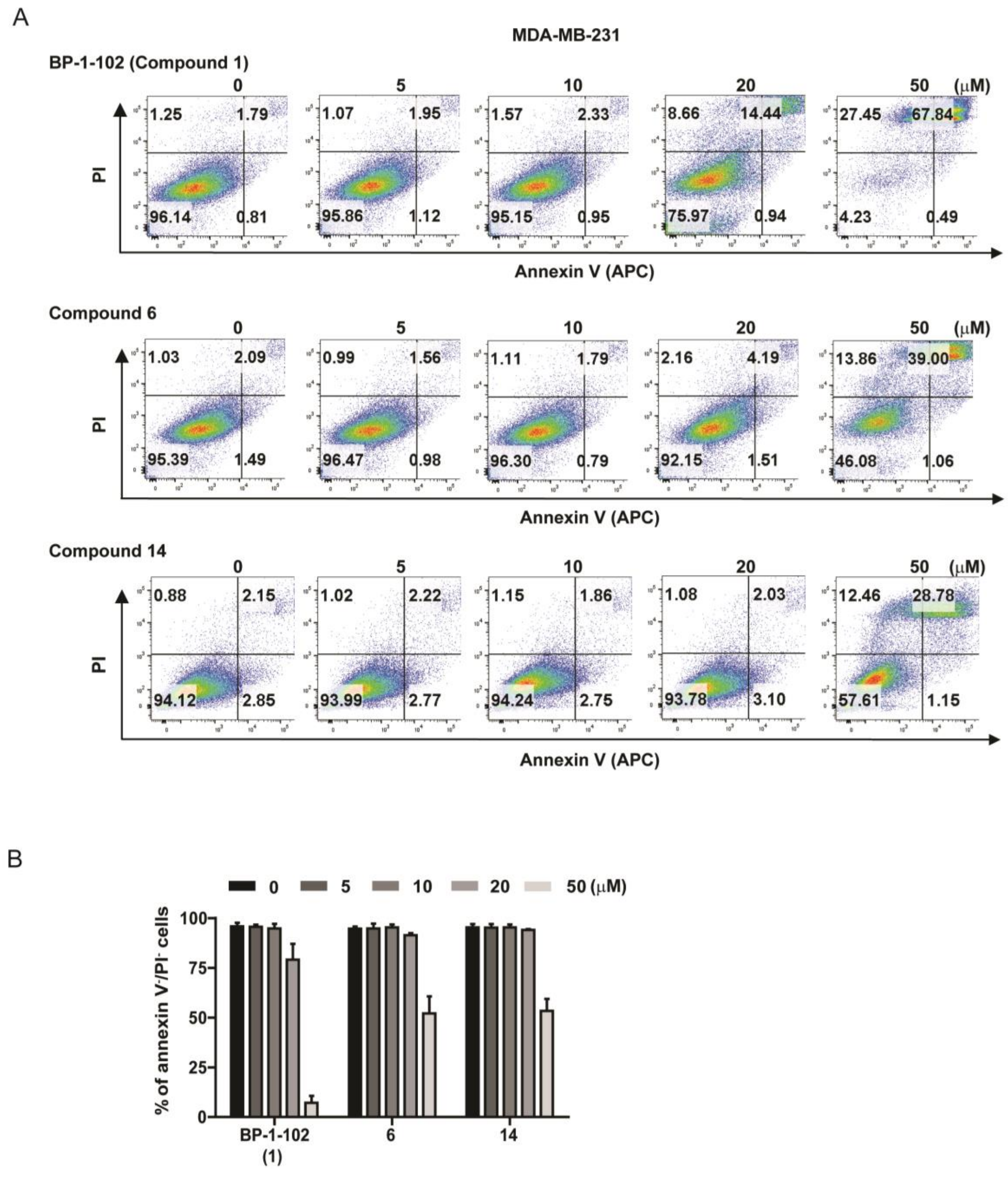

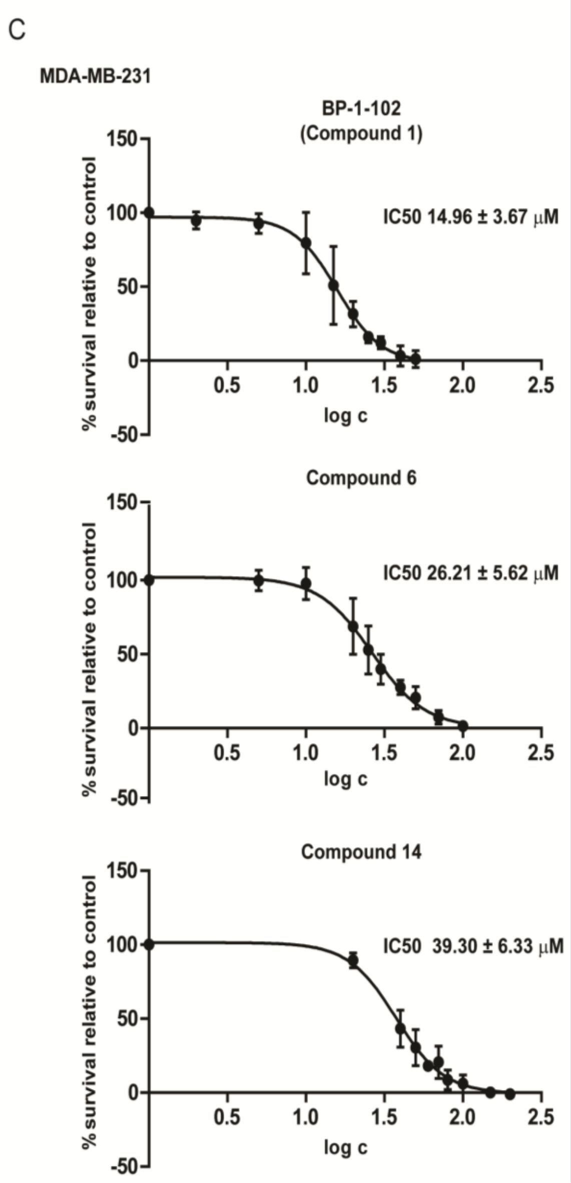

Figure S15 MDA-MB-231 cells were treated with BP-1-102 (**1**) and two newly synthesized compounds **6** and **14** at given concentrations for 24 hours. Pooled adherent and non-adherent fractions were stained with annexin V-APC and propidium iodide (PI) and analyzed by FACS. Representative scatterplots are shown in (A), percentage of viable cells (annexin V-negative/PI-negative) is plotted in (B) as mean ± S. D. (n = 2). (C) MDA-MB-231 cells were treated with BP-1-102 (**1**) and two newly synthesized compounds **6** and **14** at given concentrations for 24 hours, and the resazurin assay was performed. Data were normalized to control/untreated samples and plotted as mean ± S. D. (n ≥ 3). IC50 was calculated from each experiment using the nonlinear regression and is stated as mean ± S. D. (n ≥ 3).

3. Screening of BP-1-102 analogs and their cytotoxic effect on mouse TC-1 and TRAMP-C2 cells

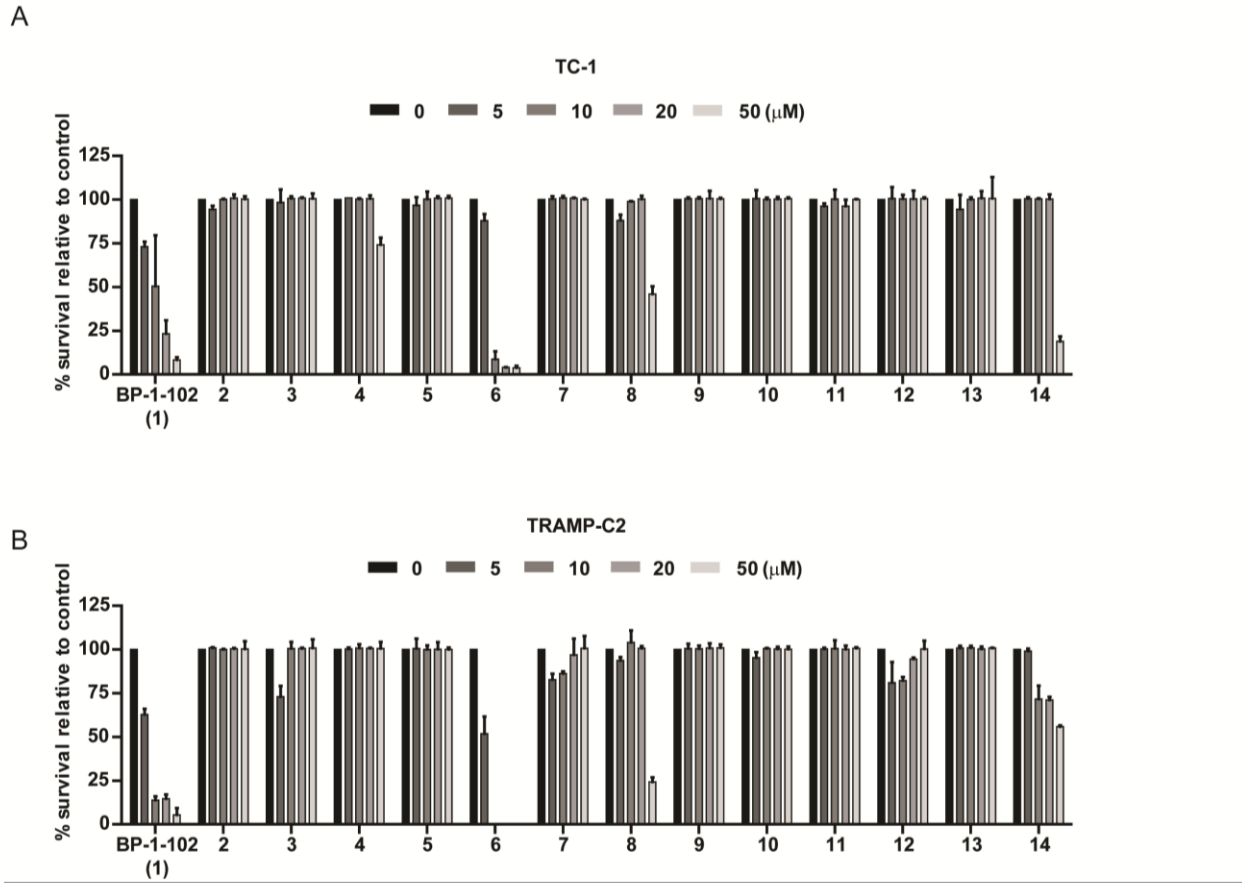

Figure S16 Cytotoxic effect of BP-1-102 analogs (concentration range 0 – 50 μM, 24 hours) on mouse TC-1 (A) and TRAMP-C2 (B) cell lines was tested by the MTT assay. Data were normalized to control samples and plotted as mean ± S.D. (n = 2).

4. Fluorescence-activated flow cytometry (FACS) analysis of apoptosis induced by selected compounds in TC-1 cells

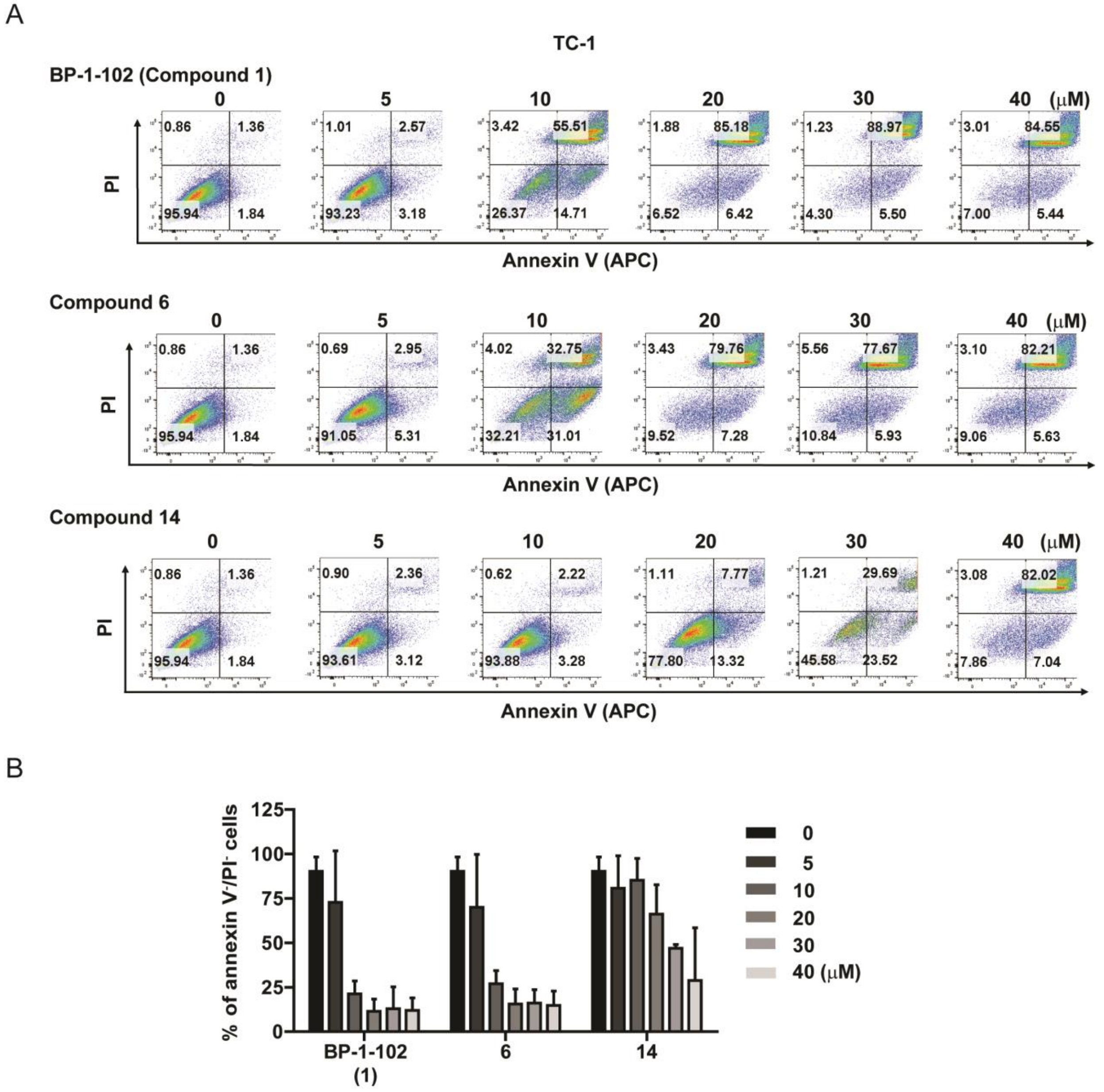

Figure S17 TC-1 cells were treated with BP-1-102 (**1**) and two newly synthesized compounds **6** and **14** at given concentrations for 24 hours. Pooled adherent and non-adherent fractions were stained with annexin V-APC and PI and analyzed by FACS. Representative scatterplots are shown in (A), percentage of viable cells (annexin V-negative/PI-negative) is plotted in (B) as mean ± S.D. (n = 2).

5. Fluorescence-activated flow cytometry (FACS) analysis of apoptosis induced by selected compounds in TRAMP-C2 cells

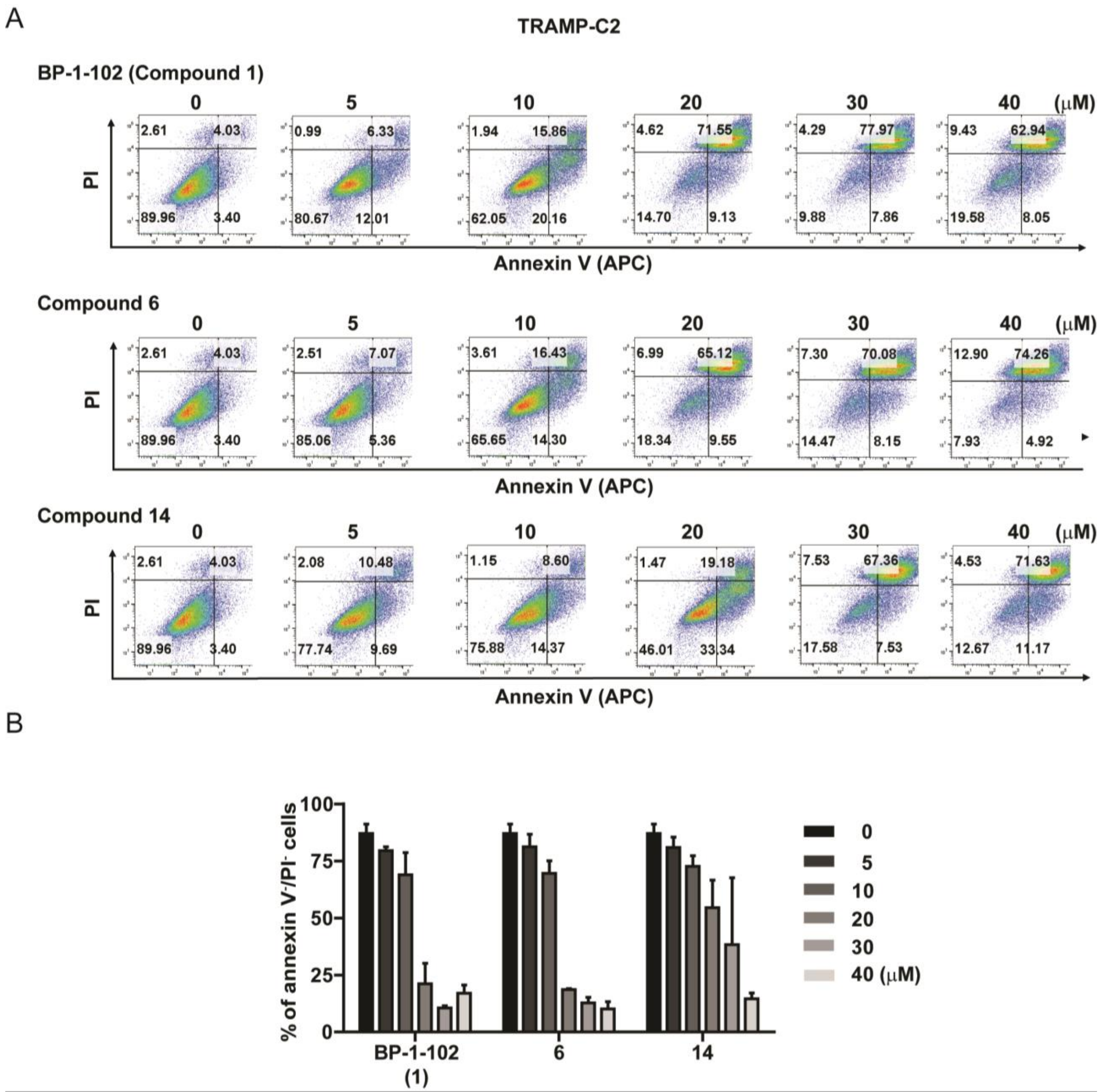

Figure S18 TRAMP-C2 cells were treated with BP-1-102 (**1**) and two newly synthesized compounds **6** and **14** at given concentrations for 24 hours. Pooled adherent and non-adherent fractions were stained with annexin V-APC and PI and analyzed by FACS. Representative scatterplots are shown in (A), percentage of viable cells (annexin V-negative/PI-negative) is plotted in (B) as mean ± S.D. (n = 2).
